# Profiling of RNA-binding protein binding sites by in situ reverse transcription-based sequencing

**DOI:** 10.1038/s41592-023-02146-w

**Published:** 2024-01-10

**Authors:** Yu Xiao, Yan-Ming Chen, Zhongyu Zou, Chang Ye, Xiaoyang Dou, Jinjun Wu, Chang Liu, Shun Liu, Hao Yan, Pingluan Wang, Tie-Bo Zeng, Qinzhe Liu, Jingyi Fei, Weixin Tang, Chuan He

**Affiliations:** 1 Department of Chemistry, The University of Chicago, Chicago, IL USA; 2 Department of Biochemistry and Molecular Biology, The University of Chicago, Chicago, IL USA; 3 Institute for Biophysical Dynamics, The University of Chicago, Chicago, IL USA; 4 Howard Hughes Medical Institute, Chicago, IL USA

**Keywords:** RNA sequencing, RNA

## Abstract

RNA-binding proteins (RBPs) regulate diverse cellular processes by dynamically interacting with RNA targets. However, effective methods to capture both stable and transient interactions between RBPs and their RNA targets are still lacking, especially when the interaction is dynamic or samples are limited. Here we present an assay of reverse transcription-based RBP binding site sequencing (ARTR-seq), which relies on in situ reverse transcription of RBP-bound RNAs guided by antibodies to identify RBP binding sites. ARTR-seq avoids ultraviolet crosslinking and immunoprecipitation, allowing for efficient and specific identification of RBP binding sites from as few as 20 cells or a tissue section. Taking advantage of rapid formaldehyde fixation, ARTR-seq enables capturing the dynamic RNA binding by RBPs over a short period of time, as demonstrated by the profiling of dynamic RNA binding of G3BP1 during stress granule assembly on a timescale as short as 10 minutes.

## Abstract (web summary)

This work introduces ARTR-seq for in situ measurement of RNA-binding protein (RBP) binding sites, which has been demonstrated in a small number of cells and for capturing dynamic RBP binding within short timeframes.

## Main

RBPs dynamically interact with their RNA targets to regulate RNA fate in all aspects, including transcription, splicing, modification, localization, translation and degradation^1^. The dysfunction of RBPs or their binding to RNA substrates can lead to various defects or even diseases. Effective methods to capture RBP–RNA interactions, particularly dynamic or even transient interactions, are critical for a better understanding of RBP and its functional effect on target RNAs^2^.

The widely used approaches to identify RBP targets are based on immunoprecipitation (IP) of the specific RBP along with its bound RNAs, either through direct RNA IP (RIP) or crosslinking IP (CLIP) assisted by covalent capture^3–15^. Substrate RNAs bound by a specific RBP can be enriched through either RIP or CLIP using the antibody against the RBP, followed by high-throughput sequencing (seq) to profile RBP targets across the whole transcriptome. CLIP-seq captures RBP binding sites on substrate RNAs via covalent crosslinking. RNase treatment digests RBP-free regions of RNAs, increasing the resolution of binding site detection^7–10,14,15^. CLIP-seq variants such as PAR-CLIP or eCLIP improve the crosslinking efficiency, specificity or binding site resolution^7,9^. While effective and widely used, these methods also have limitations. They often require a large amount of starting materials due to the low IP efficiency; the ultraviolet (UV) crosslinking in CLIP-based methods is a low-efficiency chemical reaction. Recently reported tRIP-seq and LACE-seq can be applied in low-input samples but at the cost of reducing the library complexity^12,13^.

TRIBE and STAMP type approaches fuse RBPs with an RNA base editor to introduce mutations nearby RBP binding sites, bypassing IP to identify RBP binding sites^16–21^. These methods could be readily applied to study RBP binding in live cells and with limited materials down to single-cell level. Their deployments into research have offered new opportunities; however, these editing-based methods still have limitations. They require genome manipulation by inserting base editing proteins in germlines or cell lines, hindering their application in primary cells and tissues. Inducing editing protein expression typically takes roughly 24 hours or longer, which cannot be applied to monitor dynamic RNA binding by RBPs. These base editors have their own sequence preferences, potentially changing the native binding profile of the target RBP. While we were working on our method, RT&Tag, a method derived from the CUT&Tag strategy, was published^22,23^. This method profiles RBP–RNA interaction by oligo(dT) primer-initiated reverse transcription (RT) and Tn5 tagmentation of the resulting full-length RNA–complementary DNA (cDNA) heteroduplex in isolated nuclei. RT&Tag can identify RBP binding in polyadenylated RNAs but is ineffective in nonpolyadenylated RNAs and cytoplasmic RBP binding. Due to the low efficiency of the Tn5 enzyme on heteroduplex, it requires 25,000–100,000 nuclei to obtain sufficient transcriptome-wide binding signals.

To overcome the limitations of existing methods, we introduce an assay of RT-based RBP binding site sequencing (ARTR-seq) to capture RBP–RNA interactions through in situ RT. We demonstrate that ARTR-seq sensitively profiles RBP targets with good sequencing quality, using as few as 20 cells or a single tissue section. Additionally, an imaging step can be readily built into the ARTR-seq procedure, providing direct spatial information of RBPs. With ARTR-seq, we show distinct binding patterns of splicing factors and the YTH family reader proteins of RNA *N*^6^-methyladenosine (m^6^A) modification. ARTR-seq unbiasedly detects RNA binding by RBPs in both cytoplasm and nucleus and measures RBP binding strength on RNA substrates. Furthermore, ARTR-seq could monitor dynamic RNA binding by G3BP1 during stress granule (SG) assembly on a small timescale of 10 minutes.

## Results

### Strategy and development of ARTR-seq

**Fig. 1:**
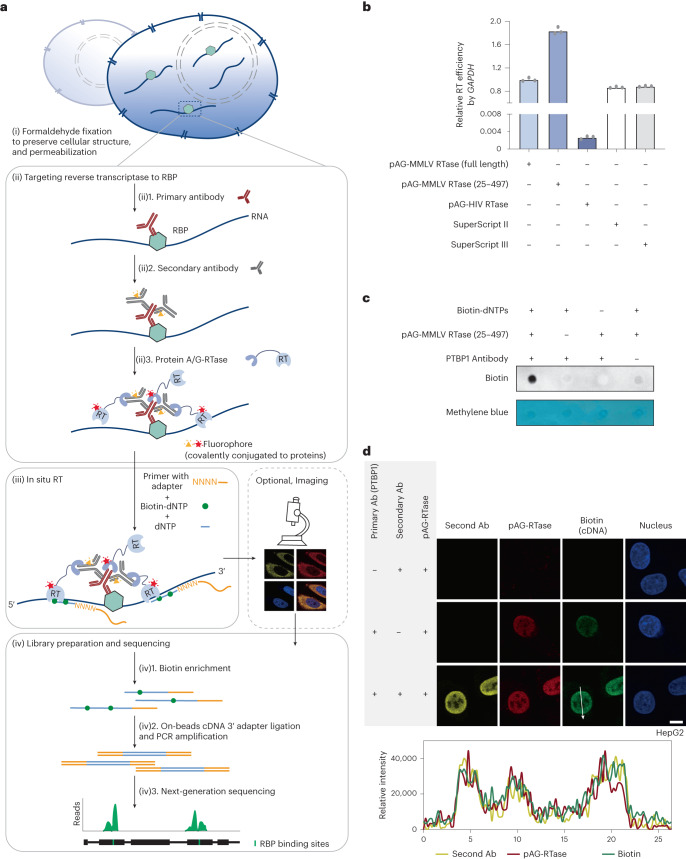
ARTR-seq strategy and validation. **a**, Scheme of ARTR-seq. **b**, RT–qPCR analysis showing the RT activity of tested purified pAG-RTase fusion proteins. Two commercial RTases, SuperScript II and SuperScript III, were loaded as positive controls. *n* = 3 biological replicates. **c**, Biotin dot blot assay showing biotinylated cDNA products produced from ARTR-seq. Methylene blue staining was the loading control. **d**, Immunofluorescence imaging of the secondary antibody (secondary Ab; yellow), pAG-RTase (red), biotinylated cDNA (green) and nucleus (blue) for PTBP1 ARTR-seq. The line graph analysis shows relative fluorescence intensity along the line. Scale bar, 10 μm. Source data

In ARTR-seq, we started with rapid formaldehyde fixation to preserve the cellular structure, followed by permeabilization of cell membranes (Fig. 1a(i)). We then targeted the reverse transcriptase (RTase) to the RBP of interest using corresponding antibodies (Fig. 1a(ii)). This involved delivering the primary antibody for RBP recognition (Fig. 1a(ii)1), followed by a secondary antibody to enhance the local antibody concentration, capitalizing on the potential for multiple secondary antibodies to bind a single primary antibody (Fig. 1a(ii)2). Subsequently, a fusion protein of protein A/G and RTase (pAG-RTase) was delivered to bind both primary and secondary antibodies, enabling site-specific attachment of RTase to the target RBP (Fig. 1a(ii)3). Each step was followed by thorough washing to remove any unbound antibodies or pAG-RTase.

**Extended Data Fig. 1:**
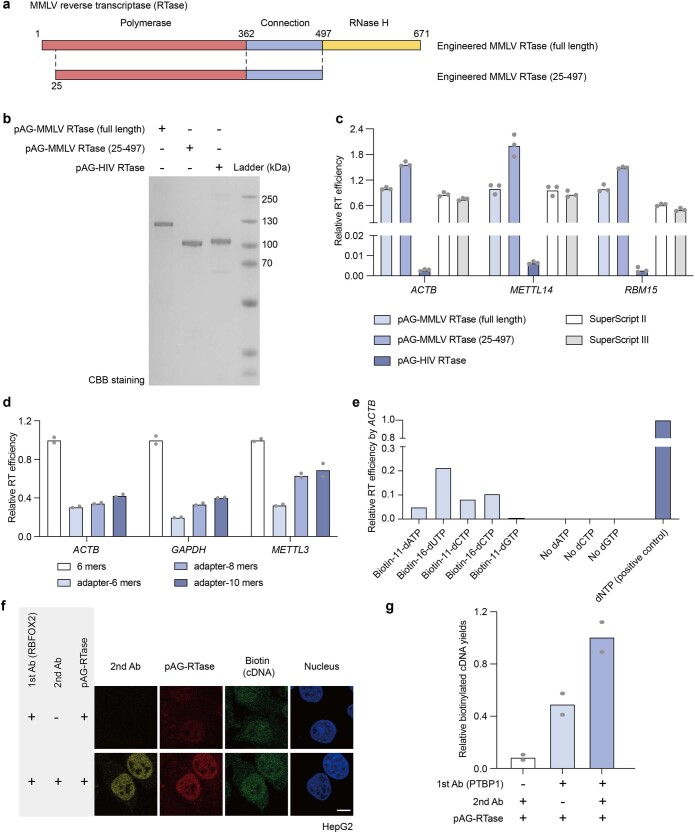
ARTR-seq setup and condition optimization. **a**, Functional domains of the engineered MMLV RTase (H8Y, D200N, T306K, W313F, T330P, D524G, L603W)^24,25^. The MMLV RTase (full length) is composed of three domains: polymerase (red), connection (purple) and RNase H (yellow). The RNase H domain and the first 24 N-terminal residues were omitted in MMLV RTase (25-497) to improve its RT activity^71^. **b**, Coomassie bright blue staining of three purified pAG-RTase fusion constructs with a link length of 30 amino acids. **c**, qRT-PCR analysis for *ACTB*, *METTL14* and *RBM15* showing the relative RT activity of three tested purified pAG-RTase fusion proteins. Two commercial RTases SuperScript II and SuperScript III were loaded as positive controls. *n* = 3 biological replicates. **d**, qRT-PCR showing the relative RT efficiency using the indicated random primers. pAG-MMLV RTase fusion protein (25-497) was used in this analysis. *n* = 2 biological replicates. **e**, qRT-PCR showing the effects of different biotinylated dNTPs on the relative RT efficiency using pAG-MMLV RTase (25-497). Biotin-16-dUTP and biotin-16-dCTP exhibited the least hindrance to RT efficiency. Both were used in the ARTR-seq procedure by mixing with regular dTTP and dCTP at a 1:1 ratio. *n* = 1 biological replicate. **f**, Immunofluorescence (IF) imaging of the secondary antibody (2nd Ab, yellow), pAG-RTase (red), newly synthesized cDNA (green), and nucleus (blue) for RBFOX2 ARTR-seq. Scale bars, 10 μm. **g**, qPCR analysis to measure relative cDNA yields of ARTR-seq samples. *n* = 2 biological replicates. Source data

After localizing RTase to the RBP, we initiated in situ RT at RBP binding sites by adding necessary RT components (Fig. 1a(iii)). To achieve efficient RT, we screened three commonly used RTases, including engineered Moloney murine leukemia virus (MMLV) RTase^24,25^, human immunodeficiency virus RTase and a truncated version of engineered MMLV RTase (25–497) in the pAG-RTase fusion constructs with a 30-amino-acid linker (Extended Data Fig. 1a,b). By employing RT with quantitative polymerase chain reaction (RT–qPCR), we confirmed pAG-MMLV RTase (25–497) as the most active and selected it for subsequent studies (Fig. 1b and Extended Data Fig. 1c).

To identify all RBP binding sites without sequence bias, we applied random RT primers with an adapter tag for library construction, and extended the primer length from commonly used 6 nucleotides (nts) to 10 nts to enhance RT efficiency (Extended Data Fig. 1d). For effective cDNA enrichment, biotinylated dNTPs were introduced into cDNA products. After screening, we found that biotin-16-dUTP and biotin-16-dCTP exhibited the least hindrance on RT efficiency (Extended Data Fig. 1e). These were included in a 1:1 ratio with regular dTTP and dCTP, respectively, in the current ARTR-seq protocol. Following cDNA enrichment with streptavidin beads, we performed adapter ligation, library amplification and high-throughput sequencing to acquire the RBP binding profile (Fig. 1a(iv)). Note that after in situ RT, immunofluorescence imaging could be performed to reveal RBP subcellular localization without disturbing the subsequent library construction if the secondary antibody and pAG-RTase are fluorophore-modified.

### Validation of ARTR-seq using PTBP1

To evaluate ARTR-seq, we applied ARTR-seq to PTBP1, a well-studied splicing factor with a variety of published CLIP-seq datasets for comparison. To verify the production of biotinylated cDNAs from in situ RT, we monitored the biotin group in the cDNA products by dot plot, confirming the incorporation of biotin and requirements of pAG-RTase and primary antibody for successful cDNA synthesis (Fig. 1c). With immunofluorescence staining, we further validated the colocalization of pAG-RTase, the secondary antibody and newly synthesized cDNA, and their signals largely disappeared on exclusion of the primary antibody, supporting the localized RT reaction performed by pAG-RTase tethered to the targeted RBP (Fig. 1d and Extended Data Fig. 1f). Note that the use of the secondary antibody increased the biotinylated cDNA yield (Fig. 1d and Extended Data Fig. 1f,g). Altogether, ARTR-seq specifically and effectively reverse transcribes RNAs near the targeted protein into biotinylated cDNA products.

**Fig. 2:**
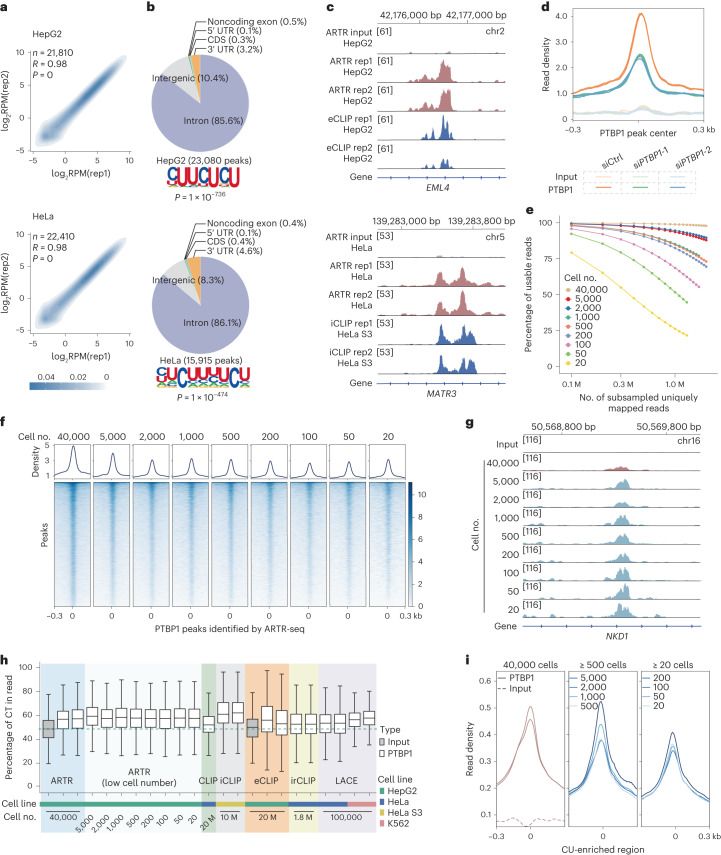
ARTR-seq captures binding sites of RBPs using as few as 20 cells. **a**, ARTR-seq replicate correlations for usable reads per gene normalized to coverage (reads per million reads mapped, RPM) for PTBP1 in HepG2 (top) and HeLa (bottom) cells, respectively. Usable reads were the remaining genomic uniquely mapped reads after deduplication. The color scale shows the point density. The coefficient *R* and *P* values were given by the two-tailed Pearson’s correlation. **b**, Peaks distribution in 3′ UTR, CDS, 5′ UTR, noncoding exon, intergenic region and intron, and the corresponding motifs of PTBP1 binding peaks identified by ARTR-seq in the HepG2 (top) and HeLa (bottom) cells, respectively. *P* values were calculated by the two-tailed binomial test in the HOMER suite^52^. **c**, Snapshots from the IGV showing the signal overlaps between ARTR-seq and eCLIP^28^ (top) or iCLIP^27^ (bottom). The ARTR-seq input was pooled by three biological replicates. **d**, ARTR-seq read density at PTBP1 binding peaks of control (siCtrl) and *PTBP1* knockdown (si*PTBP1*) HepG2 cells revealed by ATAR-seq. **e**, Percentages of usable reads in subsampled uniquely mapped reads from PTBP1 ARTR-seq with different numbers of cells. The plot shows replicate 1 for simplicity. **f**, Signal profiles and heatmaps of read density in ARTR-seq libraries constructed from 20 to 40,000 HepG2 cells at ARTR-seq-identified PTBP1 peaks. **g**, A snapshot from IGV showing the stable ARTR-seq signal in sequencing libraries constructed from different numbers of HepG2 cells. **h**, A box plot comparing the CT percentages of usable reads from libraries constructed by using ARTR-seq, CLIP^26^, iCLIP^27^, eCLIP^28^, irCLIP^10^ and LACE-seq^13^, respectively. The green dashed line represents the median percentage in the ARTR-seq input library. The sample sizes are summarized in Supplementary Table 3. **i**, Signal profiles of ARTR-seq read density at CU-enriched regions. CU-enriched regions are defined as 80 nt-wide regions with a percentage of CT content greater than 70% located in the protein-coding genes.

**Extended Data Fig. 2:**
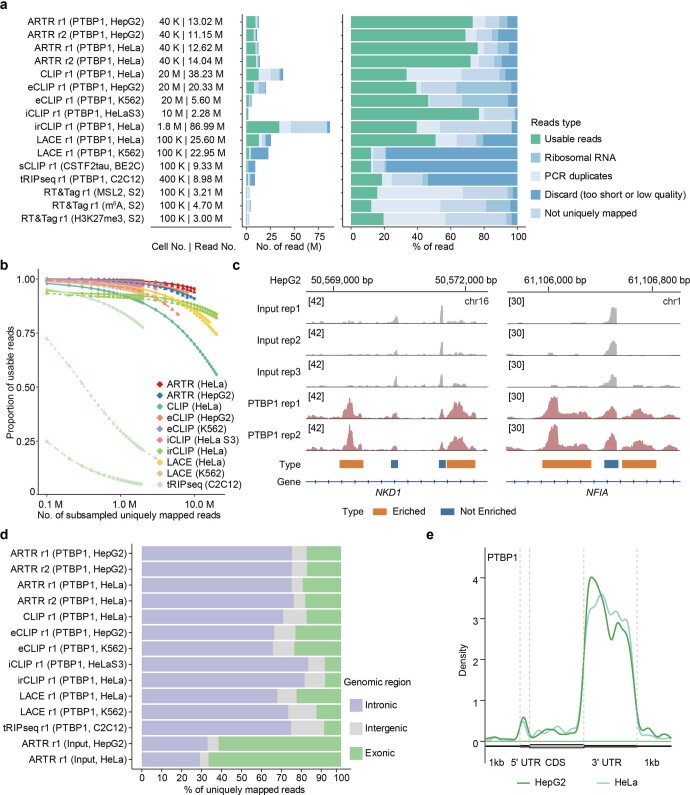
Reads comparison between ARTR-seq and other methods. **a**, Bar plots showing numbers (left) and percentages (right) of the usable reads and reads filtered after each processing step for libraries constructed by using ARTR-seq, CLIP, eCLIP, iCLIP, irCLIP, LACE-seq, sCLIP, tRIP-seq, and RT&Tag, respectively. The usable reads are defined as reads uniquely mapped to the genome and remained after PCR deduplication. **b**, Percentages of usable reads in subsampled uniquely mapped reads from PTBP1 libraries constructed by ARTR-seq, CLIP, eCLIP, iCLIP, irCLIP, LACE-seq, sCLIP, and tRIP-seq, respectively. **c**, Snapshots from Integrative Genomics Viewer (IGV) showing the read coverage of ARTR-seq libraries. The read coverage of each library was normalized by its respective sequencing depth. All tracks were set to the same scale. Regions with higher read coverage in PTBP1 ARTR-seq libraries compared to the input libraries represent true positive RBP binding signals (enriched, orange); regions with lower or comparable read coverage indicate background noise signals (not enriched, blue). Input libraries were applied to help filter out not-enriched regions. **d**, A bar plot showing the usable reads distribution in the intronic (purple), intergenic (grey) and exonic (green) regions for libraries constructed by using ARTR-seq, CLIP, eCLIP, iCLIP, irCLIP, LACE-seq, sCLIP, and tRIP-seq, respectively. About 30% of usable reads for the ARTR-seq input samples were located in introns. **e**, Meta distributions of PTBP1 ARTR-seq peaks along mRNA transcripts and flanking 1 kb regions.

We next tested ARTR-seq on PTBP1 using 40,000 HepG2 or HeLa cells, and compared the results with the published data from several known methods, namely CLIP, iCLIP, irCLIP, eCLIP, sCLIP, tRIP, LACE-seq and RT&Tag^9–13,22,26,27^. We observed that ARTR-seq displayed a comparable or higher percentage of usable reads compared to published methods, indicating a high complexity of the ARTR-seq libraries (Extended Data Fig. 2a,b). Then, we calculated the correlation between biological replicates (*R* = 0.98 for both HepG2 and HeLa samples), and confirmed good reproducibility of ARTR-seq (Fig. 2a).

**Extended Data Fig. 3:**
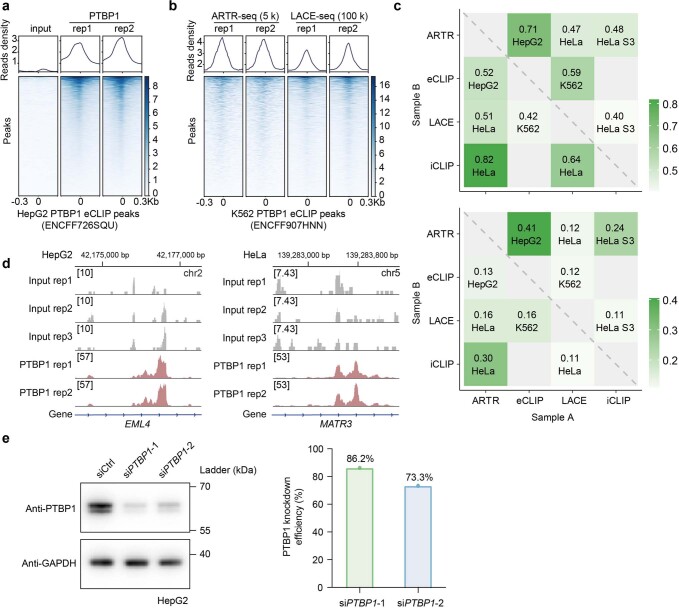
Comparison between ARTR-seq and other methods. **a**. The signal profiles and heatmaps of read density from ARTR-seq library reads at the eCLIP-identified PTBP1 peaks in HepG2 cells^28^. **b**, Signal profiles and heatmaps of read density from ARTR-seq and LACE-seq library at the eCLIP-identified PTBP1 peaks in K562 cells^13,28^. **c**, Heatmaps exhibiting the transcriptome-wide pairwise overlap of PTBP1-targeted genes (top) or peaks (bottom) among libraries from ARTR-seq, eCLIP^28^, and LACE-seq^13^ and iCLIP^27^ using the same cell line. Notably, the iCLIP data from the HeLa S3 cell line was compared with ARTR-seq using the HeLa cell line and LACE-seq using the HeLa cell line. The overlap proportion was determined as the number of detected genes (or peaks) overlapped between sample A and sample B divided by the total number of detected genes (or peaks) in sample A. The maximum gap between overlapping peaks was set at 200 nt. The overlap proportion of genes (or peaks) and the cell line of sample A were labeled in the corresponding position. **d**, IGV snapshots showing the read coverage of ARTR-seq libraries corresponding to Fig. 2c. The read coverage of each library was normalized by its respective sequencing depth. According to the ARTR-seq library types (input and PTBP1), the tracks were adjusted to distinct scales. **e**, Western blot (left) and quantification (right) displaying PTBP1 protein levels in control (siCtrl) and *PTBP1* knockdown (si*PTBP1*) HepG2 cells. GAPDH was used as an internal control for normalization. Source data

Further, we introduced input samples prepared by ARTR-seq with the omission of the primary antibody as controls to help filter out potential background signals from the nonspecific binding of the secondary antibody and RTase (Extended Data Fig. 2c). For PTBP1, we found that over 70% of usable reads and over 80% of ARTR-seq peaks were annotated to introns, with most exon peaks located within the 3′ untranslated region (3′ UTR), consistent with results reported by other methods^10,12,13,26–29^ (Fig. 2b and Extended Data Fig. 2d,e). The consensus motif of PTBP1 ARTR-seq peaks was identified as the canonical CU-enriched sequence, as known previously^30^ (Fig. 2b). At the whole-transcriptome scale, ARTR-seq reads for PTBP1 piled up at the eCLIP peaks, while the input sample did not show such accumulation^31^ (Extended Data Fig. 3a,b). Additionally, we observed that more than 50% of genes identified by ARTR-seq were also detected by other methods (52% for eCLIP, 51% for LACE-seq and 82% for iCLIP). At the peak level, ARTR-seq successfully identified 41% of eCLIP-targeted peaks (Extended Data Fig. 3c). Examination of individual PTBP1 binding sites revealed similar read distribution and density between ARTR-seq and eCLIP or iCLIP results (Fig. 2c and Extended Data Fig. 3d). To further validate PTBP1 bindings captured by ARTR-seq, we knocked down *PTBP1* in HepG2 cells using two distinct small-interfering RNAs (siRNAs) and performed ARTR-seq (Extended Data Fig. 3e). The reads located around the ARTR-seq peaks reduced accordingly on *PTBP1* knockdown, indicating the high specificity of ARTR-seq (Fig. 2d).

### Direct versus indirect binding sites detected by ARTR-seq

**Extended Data Fig. 4:**
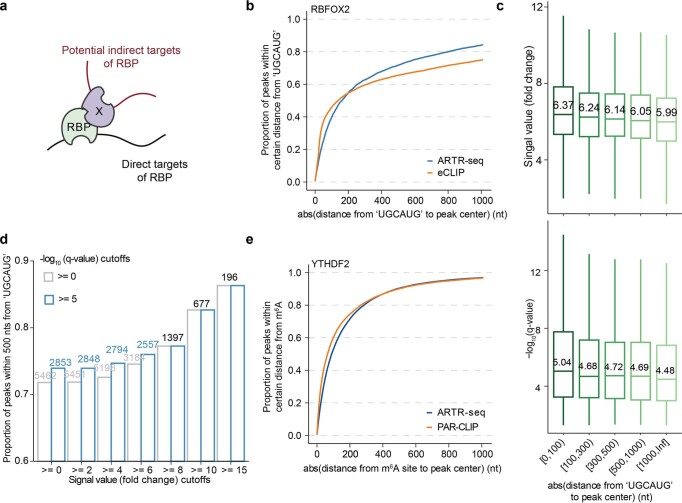
The direct versus indirect binding in ARTR-seq. **a**, A schematic diagram illustrating the simplified direct and indirect targets of the RNA binding protein (RBP). The symbol “X” represents the interacting protein or complex of the RBP. **b**, Cumulative curves displaying the proportion of RBFOX2 peaks with peak centers located within a certain absolute distance on the genome from the nearest RBFOX2 canonical motif ‘UGCAUG’ for both ARTR-seq and eCLIP^28^ (ENCODE: ENCFF871NYM). **c**, Boxplots showing ARTR-seq peaks exhibiting reduced signal values (top) and q-values (bottom) as the absolute distance to the nearest ‘UGCAUG’ site increases. From left to right, *n* = 2164, 1263, 489, 677 and 869. **d**, A bar plot illustrating the impact of signal value cutoffs and q-value cutoffs on the proportion of RBFOX2 peaks within an absolute distance of 500 nts from the closest RBFOX2 canonical motif ‘UGCAUG’. The number of remaining peaks was labeled at the top of the bar after applying the cutoffs. **e**, Cumulative curves exhibiting the proportion of YTHDF2 peaks with peak centers located within a certain absolute distance on the transcriptome from the nearest m^6^A sites identified by m^6^A-SAC-seq^33^ for both ARTR-seq and PAR-CLIP^32^.

ARTR-seq identifies RBP binding by in situ RT, enabling the capture of RNAs directly bound by the RBP (direct targets) or potentially those spatially close to the RBP (indirect targets) (Extended Data Fig. 4a). To evaluate direct versus indirect targets, we used the splicing factor RBFOX2 as an example; RBFOX2 possesses a well-defined canonical binding motif ‘UGCAUG’^9,31^. Peaks near the UGCAUG motifs likely represent direct targets, while those farther away may indicate indirect targets. We found more than 70% of ARTR-seq peaks were within 500 nts from UGCAUG. This percentage is slightly higher than that of eCLIP^9^. The two methods were comparable when the distance from peaks to UGCAUG was within 200 nts (Extended Data Fig. 4b). It is worth noting that RBFOX2 may have other noncanonical binding sites beyond the UGCAUG motif, as suggested by the similar percentage of distant RBFOX2 eCLIP peaks from this motif. Stringent cutoffs of signal values and *q* values for peaks increased confidence in identifying the direct targets, albeit at the expense of target numbers (Extended Data Fig. 4c,d). Furthermore, we also examined YTHDF2, an m^6^A binding protein^32^. Approximately 80% of YTHDF2 ARTR-seq peaks were within 300 nts from m^6^A sites identified by m^6^A-SAC-seq^33^, comparable to that from the PAR-CLIP method^32^ (Extended Data Fig. 4e). These results indicate that the indirect interactions captured in ARTR-seq are likely limited. The percentage of direct targets identified by ARTR-seq is comparable to those observed in CLIP-based methods.

**Extended Data Fig. 5:**
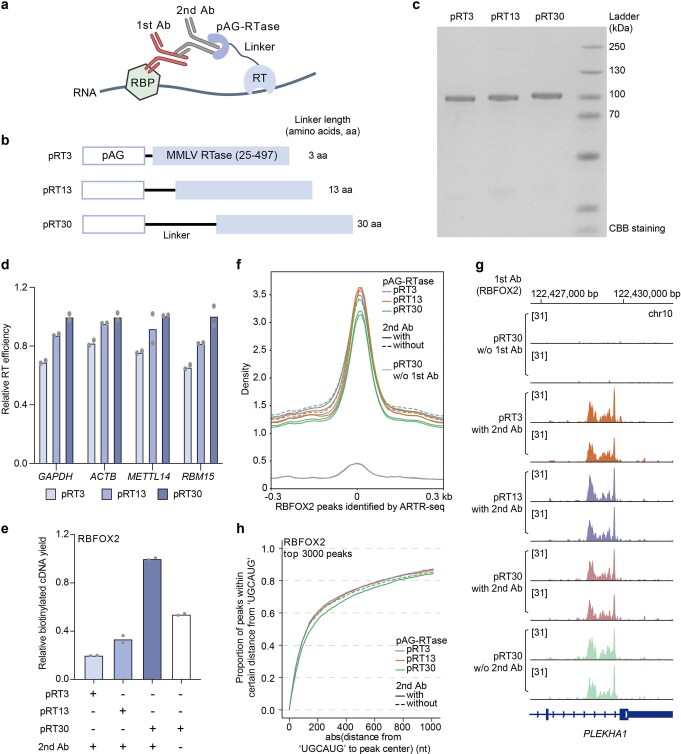
Optimizations for reducing potential indirect binding in ARTR-seq. **a**, A schematic diagram demonstrating the binding of protein A/G-reverse transcriptase (pAG-RTase), secondary antibody (2nd Ab), and the primary antibody (1st Ab). **b**, A schematic diagram showing the constructs of pAG-RTases with different amino acid (aa) linker lengths: 3 aa for pRT3, 13 aa for pRT13, and 30 aa for pRT30. **c**, Coomassie bright blue staining of the purified pRT3, pRT13 and pRT30. **d**, qRT-PCR analysis for *GAPDH*, *ACTB*, *METTL14* and *RBM15* showing the *in-vitro* RT efficiency of pAG-RTases with different linker lengths. *n* = 2 biological replicates. **e**, qPCR analysis to quantify relative cDNA yields of RBFOX2 ARTR-seq samples. *n* = 2 biological replicates. **f**, Signal profiles of ARTR-seq read density at RBFOX2 ARTR-seq peaks and flanking 0.3 kb. **g**, A snapshot from IGV showing signals of ARTR-seq libraries. **h**, Cumulative curves displaying the proportion of the top 3000 RBFOX2 peaks (with the highest signal values) with peak centers located within a certain absolute distance from the nearest ‘UGCAUG’ for ARTR-seq libraries constructed under different conditions. Source data

To further interrogate potential indirect targets identified in ARTR-seq, we limited the movement range of RTase by shortening the linker in pAG-RTase or omitting the secondary antibody (Extended Data Fig. 5a–c). We found shorter linkers reduced RT activity of pAT-RTase, indicating that shorter linkers might lead to a slowdown in the RTase kinetics (Extended Data Fig. 5d). In RBFOX2 ARTR-seq, the use of shorter linkers or omitting the secondary antibody resulted in decreased biotinylated cDNA yields but slightly increased read accumulation at RBFOX2 ARTR-seq peaks, indicating reduced RT efficiency but concentrated signals (Extended Data Fig. 5e–g). Moreover, we observed a little higher percentage (1.9–3.4%) of peaks within 500 nts of UGCAUG with a shorter linker or omitting the secondary antibody (Extended Data Fig. 5h). These findings indicate that restricting the RTase movement range tested here moderately reduced potential indirect RNAs captured by ARTR-seq. Optimal RT efficiency is another factor that needs to be considered when designing linkers.

### Resolution of ARTR-seq

**Extended Data Fig. 6:**
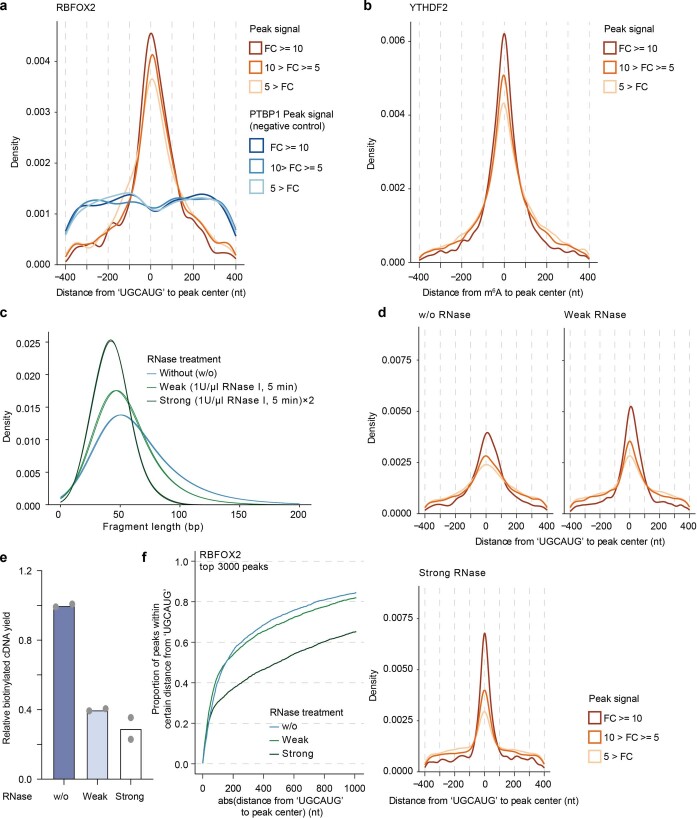
The resolution of ARTR-seq. **a**, Density plots showing the distribution of peak centers within a 400-nt window in genome flanking the RBFOX2 canonical binding motif ‘UGCAUG’ for RBFOX2 and PTBP1 (negative control) ARTR-seq libraries. **b**, Density plots showing the distribution of peak centers within a 400-nt window in transcriptome flanking m^6^A sites in HeLa cells identified by m^6^A-SAC-seq^33^ for the YTHDF2 ARTR-seq libraries. The distributions in **a**,**b** are split into three groups based on the peak signal values. **c**, Density plots exhibiting the distribution of fragment length for ARTR-seq libraries with or without RNase treatment. **d**, Density plots showing the distribution of RBFOX2 peak centers within a 400-nt window flanking the RBFOX2 canonical binding motif ‘UGCAUG’ for ARTR-seq libraries without RNase treatment, with weak RNase I treatment and with strong RNase I treatment. The distribution is split into three groups based on the peak signal values. **e**, qPCR analysis to quantify the relative cDNA yields of ARTR-seq. *n* = 2 biological replicates. **f**, Cumulative curves displaying the proportion of the top 3000 RBFOX2 peaks (with the highest signal values) with peak centers located within a certain absolute distance from the nearest ‘UGCAUG’ for ARTR-seq libraries constructed with or without RNase treatment. Source data

To assess the resolution of ARTR-seq, we examined the distribution of RBFOX2 peak centers around UGCAUG sites, and observed a clear enrichment with most peaks positioned within 200 nts flanking the UGCAUG motif (Extended Data Fig. 6a). Furthermore, we conducted a parallel analysis on YTHDF2. Compared to RBFOX2, we observed a similar but more enriched distribution for YTHDF2 around the corresponding m^6^A sites, further supporting the capability of ARTR-seq in capturing RBP binding sites (Extended Data Fig. 6b).

In an attempt to improve the resolution of binding site identification by ARTR-seq, we evaluated the impact of RNase treatment on RBFOX2 ARTR-seq. As expected, the stronger RNase treatment reduced the library fragment lengths (Extended Data Fig. 6c). We observed that the stronger RNase treatment led to a sharper enrichment of RBFOX2 ARTR-seq peaks around UGCAUG sites, indicating an improved resolution upon RNase treatment (Extended Data Fig. 6d). Through quantification of biotinylated cDNA, we found that samples with stronger RNase treatment exhibited lower RT efficiency (Extended Data Fig. 6e). Moreover, stronger RNase treatment markedly reduced the proportion of peaks located within 500 nts of the canonical UGCAUG motif. This suggests that the application of RNase may reduce reads from direct targets, thereby potentially elevating the ratio of nonspecific or indirect binding signals (Extended Data Fig. 6f). Overall, our studies revealed that RNase treatment could improve ARTR-seq resolution. The strength of RNase treatment in ARTR-seq needs to be optimized to achieve the desired balance between resolution and sensitivity, especially for samples with limited starting materials.

### ARTR-seq detects PTBP1 binding sites with as few as 20 cells

**Extended Data Fig. 7:**
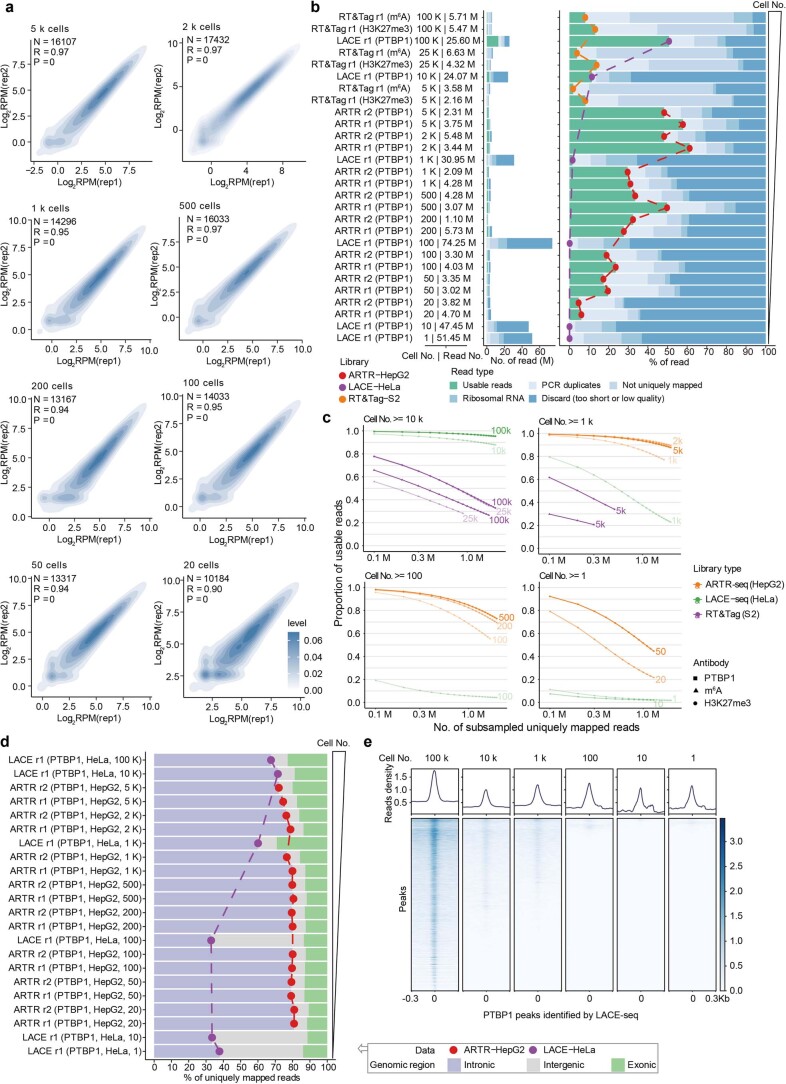
Application of ARTR-seq using low input samples. **a**, ARTR-seq replicate correlations for usable reads per gene normalized to coverage (RPM) for PTBP1 with different numbers of HepG2 cells. The color scale shows the point density. The coefficient R and P-values were given by the two-tailed Pearson’s correlation. **b**, Bar plots showing numbers (left) and percentages (right) of the usable reads and reads filtered after each processing step for libraries constructed from different cell numbers by ARTR-seq, LACE-seq^13^, and RT&Tag^22^. The libraries generated by the same method are linked with the line and indicated in the same color. **c**, Percentages of usable reads in subsampled uniquely mapped reads from PTBP1 libraries constructed from different numbers of cells by ARTR-seq, LACE-seq^13^, and RT&Tag^22^, respectively. Different methods are indicated by colors. **d**, A bar plot showing the usable reads distribution in the intronic (purple), intergenic (grey) and exonic (green) regions of libraries constructed from different numbers of cells by ARTR-seq and LACE-seq, respectively^13^. The libraries generated by the same method are linked with the line and indicated with the same color. **e**, The signal profile and heatmap of read density in LACE-seq with different numbers of cells at LACE-seq-identified PTBP1 peaks^13^.

The in situ RT-based ARTR-seq bypasses the IP step to minimize sample loss, potentially making it feasible for low cell number samples. To test this, we generated libraries for PTBP1 using different numbers of HepG2 cells and compared the results with published data from LACE-seq and RT&Tag of low cell number samples^13,22^. The correlations remained strong for ARTR-seq libraries prepared from as few as 20 cells (Extended Data Fig. 7a). Additionally, ARTR-seq libraries exhibited a much higher percentage of usable reads compared to other methods when using comparable numbers of cells (Fig. 2e and Extended Data Fig. 7b,c). Furthermore, PTBP1 ARTR-seq presented a consistently high percentage of intronic reads, suggesting its effectiveness in capturing informative reads even with the limited starting materials (Extended Data Fig. 7d). We further subsampled libraries to an equal sequencing depth and examined their reads distribution at peaks identified in the corresponding bulk samples. Compared to LACE-seq, ARTR-seq exhibited a clearer accumulation at the peak center with a higher proportion of effective reads (Fig. 2f and Extended Data Fig. 7e). Visible ARTR-seq signal remained stable for libraries with different numbers of cells as exemplified in the Integrative Genomics Viewer (IGV) plot (Fig. 2g).

Because PTBP1 binds to a canonical CU-enriched sequence, we compared the CT percentages in usable reads of PTBP1 libraries constructed by different methods. We found that all the ARTR-seq libraries showed comparable or higher CT percentages compared to that of other methods^10,13,26–28^ (Fig. 2h). We further assessed the read distribution around CU-enriched regions and observed the stable read accumulation in ARTR-seq libraries of all cell numbers, peaking at the region center (Fig. 2i). Taken together, ARTR-seq can effectively and specifically capture the RBP binding sites, even with limited starting materials.

### Application of ARTR-seq in mouse embryo sections

RBPs can have strong tissue-specific expression, or are only expressed in certain tissues rather than cultured cells. Identifying RBP binding sites in tissues remains technically challenging^34^. IP-based methods require dissociating tissues into single cells for UV crosslinking, limiting their application to whole tissues, particularly embedded frozen tissues or formalin-fixed tissues. Editing-based methods require genetic modification and cannot be applied to patient tissues.

**Fig. 3:**
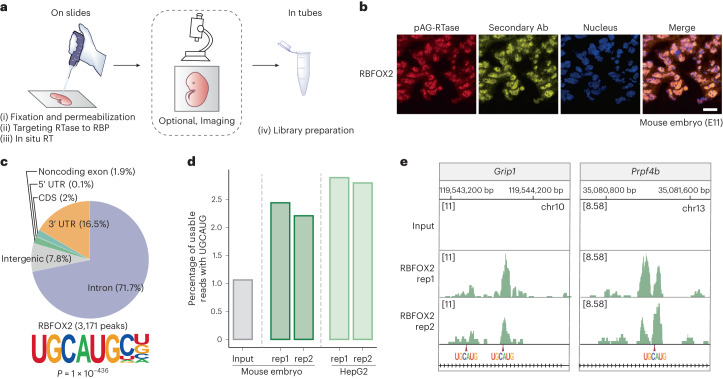
ARTR-seq maps RBP binding sites in tissues. **a**, ARTR-seq scheme for tissue samples. A section of tissue is fixed on the slide for ARTR-seq. The RTase is attached to the RBP of interest by specific antibodies and a protein A/G fusion, followed by in situ RT, with a built-in optional imaging step. The cDNA product is then collected for library preparation. **b**, Immunofluorescence imaging showing the localization of pAG-RTase (red), secondary Ab (yellow) and nucleus (blue) in the mouse embryo section (E11). Scale bar, 20 μm. **c**, Peaks distribution (top) in 3′ UTR, CDS, 5′ UTR, noncoding exon, intergenic region and intron, and motifs (bottom) of RBFOX2 binding peaks identified by ARTR-seq in the mouse embryonic tissue. *P* value was calculated by the two-tailed binomial test in the HOMER suite^52^. **d**, A bar plot showing the percentage of usable reads containing the RBFOX2 canonical UGCAUG motif for mouse embryos and HepG2 cells. **e**, Snapshots from IGV showing overlap of RBFOX2 ARTR-seq signal in mouse embryos with UGCAUG-containing sequences. The positions of the UGCAUG motifs are indicated with arrows.

ARTR-seq offers an opportunity for identifying RBP binding sites in tissues. We studied RBFOX2 with a section of OCT-embedded E11 mouse embryo to validate the feasibility of ARTR-seq in tissue samples (Fig. 3a). We first confirmed the nuclear localization of RBFOX2 with the ARTR-seq built-in imaging (Fig. 3b). The ARTR-seq reads for mouse embryo tissue showed a high percentage of usable reads and good reproducibility between biological replicates (Supplementary Fig. 1a,b). Compared to the input, a higher percentage of usable reads from RBFOX2 ARTR-seq were mapped to introns, consistent with the known binding preference of RBFOX2 (ref. ^31^) (Supplementary Fig. 1c). RBFOX2 binding peaks were mostly located in introns and contained the canonical UGCAUG motif^9^ (Fig. 3c). Additionally, we observed that mouse tissue samples displayed a similar percentage of usable reads containing UGCAUG motifs to that of HepG2 cell samples, indicating comparable signal detection efficiency of ARTR-seq for tissues and cultured cells (Fig. 3d). Examination of individual binding sites further supported the recognition of UGCAUG by RBFOX2 (Fig. 3e). Overall, ARTR-seq can identify RBP binding sites in embedded tissue samples with high specificity.

### ARTR-seq profiles regulatory features of splicing factors

**Fig. 4:**
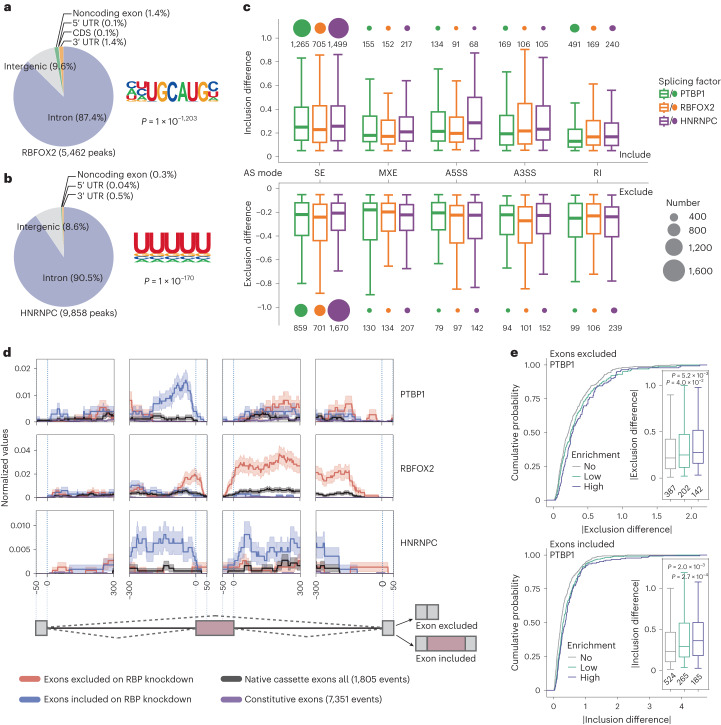
RNA binding by splicing factors identified in ARTR-seq. **a**,**b**, Peaks distribution (right) in 3′ UTR, CDS, 5′ UTR, noncoding exon, intergenic region and intron, and the corresponding motifs (left) of RBFOX2 (**a**) and HNRNPC (**b**) peaks detected by ARTR-seq in HepG2 cells. *P* values were calculated by the two-tailed binomial test in the HOMER suite^52^. **c**, Boxplots showing the splicing differences of five alternative splicing (AS) modes upon the knockdown of *PTBP1* (green), *RBFOX2* (orange) and *HNRNPC* (purple). The splicing modes include skipped exon (SE), mutually exclusive exon (MXE), alternative 5′ splice site (A5SS), alternative 3′ splice site (A3SS) and retained intron (RI). The size of circles on the top or bottom of each bar indicates event numbers. **d**, Normalized splicing maps^37^ showing the peak density for skipped exons that were excluded (red) or included (blue) upon corresponding splicing factor knockdown. Lines depict average ARTR-seq peak density. The confidence bounds show the standard errors of the alternatively included or excluded events. **e**, Cumulative curves and boxplots (inset) showing the absolute value of exon splicing differences upon *PTBP1* knockdown. PTBP1-regulated genes were divided into three groups according to their enrichment in ARTR-seq, including no enrichment (No, 0 ≤ enrichment ≤ 1), low enrichment (Low, 1 < enrichment ≤ 2) and high enrichment (High, 2 < enrichment). The sample size was labeled below the respective box. *P* values were determined by the two-tailed Student’s *t*-test of the indicated group versus the ‘no enrichment’ group.

**Extended Data Fig. 8:**
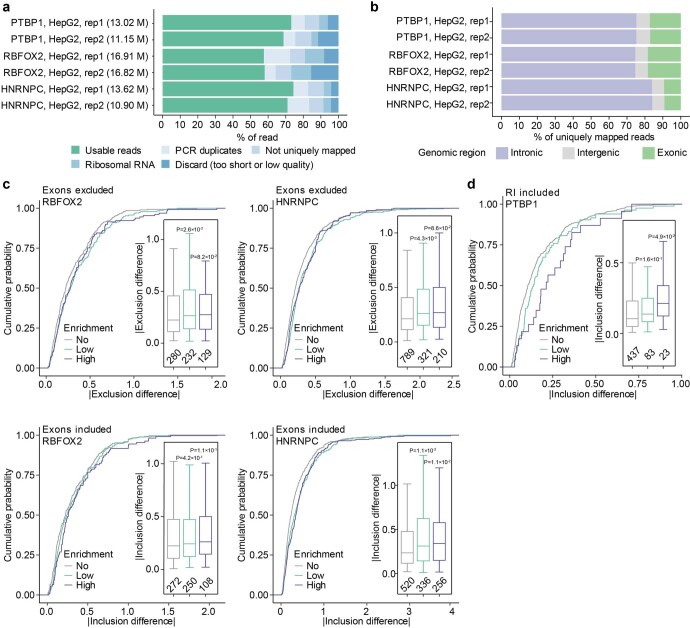
ARTR-seq-detected binding signals of splicing factors. **a**, Bar plots showing numbers (left) and percentages (right) of the usable reads and reads filtered in each processing step for ARTR-seq libraries of PTBP1, RBFOX2 and HNRNPC. **b**, A bar plot showing the usable reads distribution in the intronic (purple), intergenic (grey) and exonic (green) regions for ARTR-seq libraries of PTBP1, RBFOX2 and HNRNPC. **c**, Cumulative curves and boxplots (inside) showing the absolute value of splicing difference upon *RBFOX2* (left) or *HNRNPC* (right) knockdown. **d**, Cumulative curves and boxplots (inside) showing the absolute value of splicing differences of included RI upon *PTBP1* knockdown. In **c**,**d**, RBP-regulated genes were divided into three groups according to their enrichment in ARTR-seq, including no enrichment (No, 0 ≤ enrichment ≤ 1), low enrichment (Low, 1 < enrichment ≤ 2) and high enrichment (High, 2 < enrichment). The sample size in **c**,**d** was indicated below the respective box. P-values in **c**,**d** were determined using the two-tailed Student’s t-test of indicated group versus ‘no enrichment’ group.

PTBP1 and RBFOX2 are well-known splicing factors, with PTBP1 belonging to the heterogeneous ribonucleoprotein (hnRNP) family^35^. To show broader applicability of ARTR-seq, we also studied HNRNPC, another splicing factor belonging to the hnRNP family (Extended Data Fig. 8a). Consistent with the binding preference of the splicing factors, both reads (over 70%) and peaks (over 80%) from the ARTR-seq libraries of all three splicing factors (PTBP1, HNRNPC and RBFOX2) were mainly located in introns in HepG2 cells (Fig. 4a,b and Extended Data Fig. 8b). The RNA-binding motifs of RBFOX2 and HNRNPC were the canonical UGCAUG and U-rich sequences, respectively, consistent with the previous report^31^ (Fig. 4a,b).

To explore the association between splicing factor binding and splicing regulation, we identified the alternative splicing events by comparing the ENCODE (Encyclopedia of DNA Elements) RNA sequencing (RNA-seq) data from RBP-knockdown (KD) cells with those from control cells^36^. We found most alternative splicing events were categorized as exon skipping (Fig. 4c). We then generated ‘splicing maps’ for exon skipping events^37^ (Fig. 4d). The corresponding ARTR-seq peaks were predominantly enriched at upstream proximal introns of the included exons upon RBP-KD, at downstream proximal introns of the excluded exons upon *RBFOX2*-KD and at both upstream and downstream proximal introns of the included exons upon *HNRNPC*-KD, but not around native cassette exons and constitutive exons. We quantified relative RBP binding strength by ARTR-seq enrichment at the gene level, and observed that genes with higher ARTR-seq enrichment tend to present a higher splicing difference upon RBP-KD (Fig. 4e and Extended Data Fig. 8c). In addition to exon skipping, the number of included retained introns upon *PTBP1*-KD (491 events) outnumbered other splicing modes. With further inspection, we found that higher enrichment corresponded to higher splicing inclusion differences of retained introns, similar to the trend observed for exon skipping instances (Extended Data Fig. 8d). Altogether, ARTR-seq robustly captures distinctive binding patterns for different splicing factors, and the ARTR-seq enrichment could indicate differences in splicing.

### ARTR-seq identifies binding features of m^6^A reader proteins

In addition to sequence recognition, RBPs can also target RNAs in a chemical modification-dependent manner. m^6^A modification is the most prevalent chemical modification in mammalian messenger RNA (mRNA), and m^6^A reader proteins can preferentially bind m^6^A-modified RNAs to regulate its processing and metabolism in both the nucleus and cytoplasm^32,38–41^. We performed ARTR-seq for two cytosolic m^6^A readers YTHDF1 and YTHDF2, and a nuclear reader YTHDC1 in HeLa cells.

**Fig. 5:**
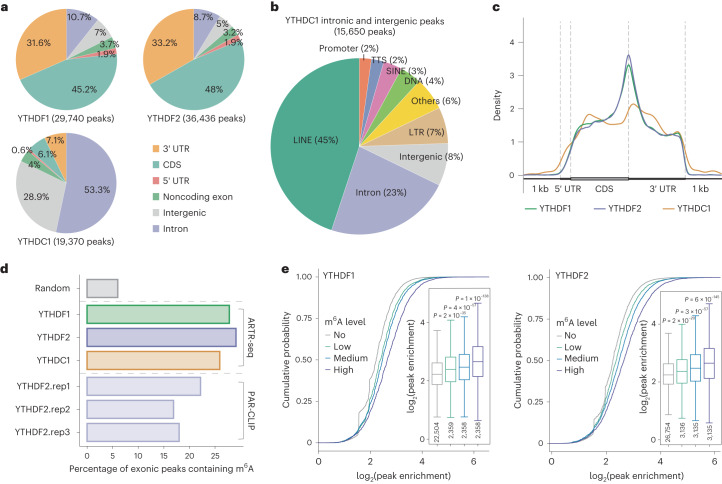
ARTR-seq maps binding features of the selected m^6^A binding proteins. **a**, Peaks distribution in 3′ UTR, CDS, 5′ UTR, noncoding exon, intergenic region and intron of YTHDF1, YTHDF2 and YTHDC1 identified by ARTR-seq for HeLa cells. **b**, A pie chart showing the detailed genomic feature distribution of YTHDC1 intronic and intergenic binding peaks. LINE, long interspersed nuclear elements. **c**, Aggregation profiles showing the meta distributions of binding peaks for YTHDF1 (green), YTHDF2 (purple) and YTHDC1 (orange) along mRNA transcripts. **d**, A bar plot showing the percentage of exonic peaks containing m^6^A sites detected by m^6^A-SAC-seq (ref. ^33^) for the m^6^A reader proteins. The random peaks are random exonic regions with the same lengths as pooled ARTR-seq peaks from the three reader proteins. Three replicates of published YTHDF2 PAR-CLIP data were used as the positive controls^32^. **e**, Cumulative curves and boxplots (inset) exhibit the log_2_ peak enrichment of ARTR-seq targets for YTHDF1 (left) and YTHDF2 (right). Peaks of m^6^A reader proteins were divided into four groups according to the modification fraction of the containing m^6^A (sum value) quantified by m^6^A-SAC-seq. The peaks without m^6^A were categorized in one group (No), and other peaks were divided into three groups with an equal number of peaks, including low m^6^A fraction (Low), medium m^6^A fraction (Medium) and high m^6^A fraction (High). The sample size was indicated below the respective box. *P* values were determined by the two-tailed Student’s *t*-test of indicated group versus the ‘no m^6^A’ group.

**Extended Data Fig. 9:**
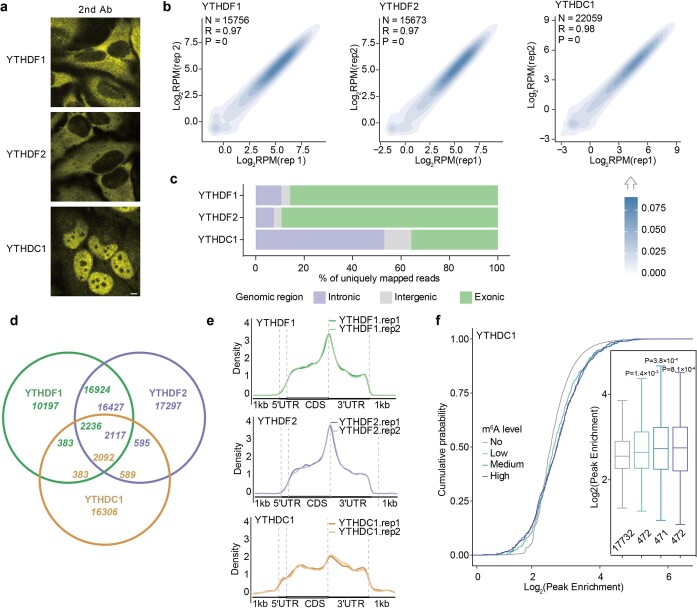
Binding features of m^6^A reader proteins detected by ARTR-seq. **a**, IF imaging showing the subcellular localization of YTHDF1, YTHDF2, and YTHDC1 in HeLa cells. Scale bars, 5 μm. **b**, ARTR-seq replicate correlations for usable reads per gene normalized to coverage (RPM) for YTHDF1, YTHDF2, and YTHDC1. The color scale shows the point density. The coefficient R and P-values were given by the two-tailed Pearson’s correlation. **c**, Distribution of usable reads in the intronic (purple), intergenic (grey) and exonic (green) regions for ARTR-seq libraries of the individual m^6^A binding proteins. **d**, Venn plot illustrating overlap of peaks identified by ARTR-seq for YTHDF1, YTHDF2 and YTHDC1. **e**, Aggregation profiles showing the meta distributions of binding peaks along mRNA transcript detected in two biological ARTR-seq replicates for YTHDF1 (green), YTHDF2 (purple), and YTHDC1 (orange). **f**, Cumulative curves and boxplots (inside) showing the log_2_ peak enrichment of ARTR-seq targets for YTHDC1. YTHDC1 peaks were divided into four groups according to the modification fraction of the containing m^6^A (sum value) quantified by m^6^A-SAC-seq^33^. The peaks without m^6^A were categorized in one group (No), and other peaks were divided into three groups with an equal number of peaks, including low m^6^A fraction (Low), medium m^6^A fraction (Medium) and high m^6^A fraction (High). The sample size was indicated below the respective box. P-values were determined by the two-tailed Student’s t-test of indicated group versus ‘no m^6^A’ group.

We first verified the subcellular localization of the three readers with ARTR-seq built-in imaging (Extended Data Fig. 9a). Sequencing data from ARTR-seq remained highly reproducible between replicates (Extended Data Fig. 9b). Over 80% of the peaks of the two cytoplasmic m^6^A readers (YTHDF1 and YTHDF2) were located in exons, whereas roughly 81% of the peaks of nuclear reader YTHDC1 were located in introns or intergenic regions, consistent with their distinct subcellular localization (Fig. 5a and Extended Data Fig. 9a,c). The high unique peak ratios observed for the three reader proteins (84.2% for YTHDC1, 34.3% for YTHDF1 and 47.5% for YTHDF2) are attributed to their unique subcellular localization; YTHDF1 and YTHDF2 display different sequences of the N-terminal low-complexity domains, which most likely affect their binding to different partner proteins and therefore different RNA targets^42^ (Extended Data Fig. 9d). We further investigated the much more abundant non-exonic peaks of YTHDC1, and found more than half of them located in repeat elements, with long interspersed nuclear elements (roughly 45%) being the most prevalent, consistent with a previous report^41^ (Fig. 5b). Analysis of exonic peak distribution along mRNA showed enrichment around stop codons for all these m^6^A readers, resembling the meta profile of m^6^A modifications, especially for YTHDF1 and YTHDF2 (ref. ^33^) (Fig. 5c and Extended Data Fig. 9e).

Further, we calculated the percentage of exonic peaks overlapping with m^6^A sites in polyadenylated RNAs identified by m^6^A-SAC-seq (ref. ^33^). The ARTR-seq peaks for all three readers showed higher percentages than random peaks, comparable to the YTHDF2 peaks from PAR-CLIP^32^, supporting the m^6^A-dependent binding features of these three readers (Fig. 5d). We then analyzed the association between the m^6^A fraction and RBP binding strength, and observed that the group with higher m^6^A fractions showed higher RBP enrichment signals for YTHDF1 and YTHDF2, further suggesting ARTR-seq can measure the relative binding strength of RBPs (Fig. 5e). However, the association for YTHDC1 was weaker, potentially due to the limited number of exonic YTHDC1 peaks (Extended Data Fig. 9f). Overall, ARTR-seq captures different features of three m^6^A binding proteins in cytoplasm and nucleus.

### Dynamic RNA binding of G3BP1 during SG assembly

SGs are membraneless organelles composed of proteins and RNAs and formed in response to stress. The RBP G3BP1 is the central node in the network of protein–RNA interaction during SG assembly^43,44^. Under sodium arsenite (NaAsO_2_) treatment, SGs could be observed after 13 min with a progressive increase in size over time, with most of the SG assembly completed by 40 min, providing a rapid stress response^45^. However, whether RNA targets of G3BP1 vary during SG assembly has yet to be investigated.

**Fig. 6:**
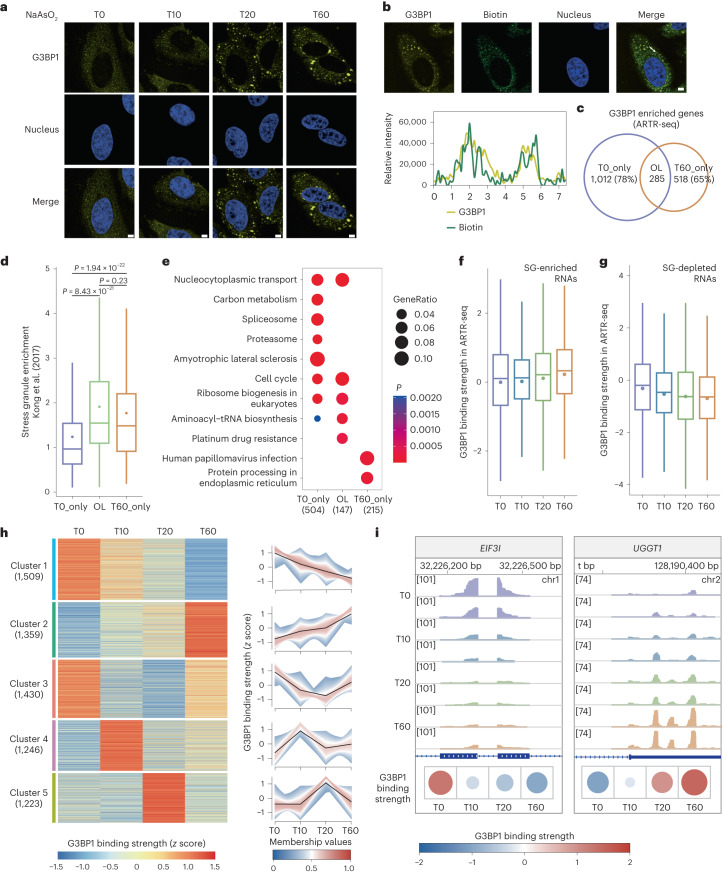
Dynamic RNA binding of G3BP1 during the assembly of SGs. **a**, Immunofluorescence imaging showing the localization of G3BP1 in HeLa cells without treatment (T0) and with 0.5 mM NaAsO_2_ treatment for 10 min (T10), 20 min (T20) and 60 min (T60), respectively. Scale bars, 5 μm. **b**, Immunofluorescence imaging (top) showing that G3BP1 (yellow) was colocalized with biotinylated cDNA (green) generated from ARTR-seq. The line graph analysis (bottom) shows the relative fluorescence intensity along the line. Scale bar, 5 μm. **c**, A Venn diagram showing the overlap between the G3BP1–RNA targets at T0 and T60. **d**, A box plot exhibiting SG enrichment of RNA targets from three groups defined in **c**, including T0 only (T0_only, *n* = 965) fraction, T0 and T60 overlapped (OL, *n* = 274) fraction and T60 only (T60_only, *n* = 482) fraction. SG enrichment values were reported in SG RNA-seq^46^. *P* values were determined by the two-tailed Wilcoxon test. **e**, KEGG enrichment analysis showing RNA targets from three groups are enriched in distinct pathways. *P* values were calculated by the clusterProfiler package^53^ using the one-tailed hypergeometric test. **f**,**g**, Boxplots of G3BP1 binding strength for SG-enriched RNAs (*n* = 1,512, **f**) and SG-depleted RNAs (*n* = 1,671, **g**). G3BP1 binding strength was defined as ARTR-seq reads log_2_FC(G3BP1/input). SG-enriched RNAs and SG-depleted RNAs were obtained from a previous SG RNA-seq report^46^. **h**, A heatmap (left) depicting changing patterns of G3BP1 binding strength for RNA clusters across time. RNAs were ranked from large to small according to the s.d. of G3BP1 binding intensity over different time intervals, and the top 50% of RNAs were selected and clustered by fuzzy *c*-means. Line plots (right) exhibit the corresponding change of G3BP1 binding strength in each cluster. Each line represents one gene, with the black line being the centroid of the cluster. **i**, IGV snapshots showing two G3BP1–RNA targets with decreased (left) and increased (right) binding strength, and each panel was normalized by counts per million. Heatmaps (bottom) show G3BP1 binding strength with the size of circles representing its absolute value.

Taking advantage of the potential high temporal resolution offered by fast formaldehyde fixation and low material requirements of ARTR-seq, we performed ARTR-seq for G3BP1 in HeLa cells with 0.5 mM NaAsO_2_ treatment and monitored the SG assembly process at time intervals of 0, 10, 20 and 60 min poststress. We first visualized G3BP1 localization using immunofluorescence imaging, and confirmed the gradual condensation of G3BP1 into granules over time (Fig. 6a). The colocalization of G3BP1 and biotinylated cDNA products was further verified (Fig. 6b). Subsequently, the verified samples were used for ARTR-seq library construction and sequencing. We determined G3BP1 binding strength by calculating the ARTR-seq log_2_ fold change (log_2_FC) between G3BP1 and input samples at the gene level. Roughly 78% of G3BP1–RNA targets (log_2_FC ≥ 1, *P* < 0.05) were no longer enriched at 60 min (T60) post-NaAsO_2_ treatment (Fig. 6c). SG enrichment of RNA was previously assessed by sequencing RNAs isolated from NaAsO_2_-induced SGs to quantify their relative localization within SGs^46^. Through integrative analysis, we observed that G3BP1 targets at T60 showed notably higher SG enrichment compared to the starting point without stress (Fig. 6d). These results support the accuracy of ARTR-seq and revealed distinct RNA binding of G3BP1 in the presence and absence of stress. The functions of stress-induced G3BP1 targets (T60_only) were enriched to Kyoto Encyclopedia of Genes and Genomes (KEGG) pathways of protein processing in the endoplasmic reticulum and human papillomavirus infection, consistent with previous observations^47,48^ (Fig. 6e).

**Extended Data Fig. 10:**
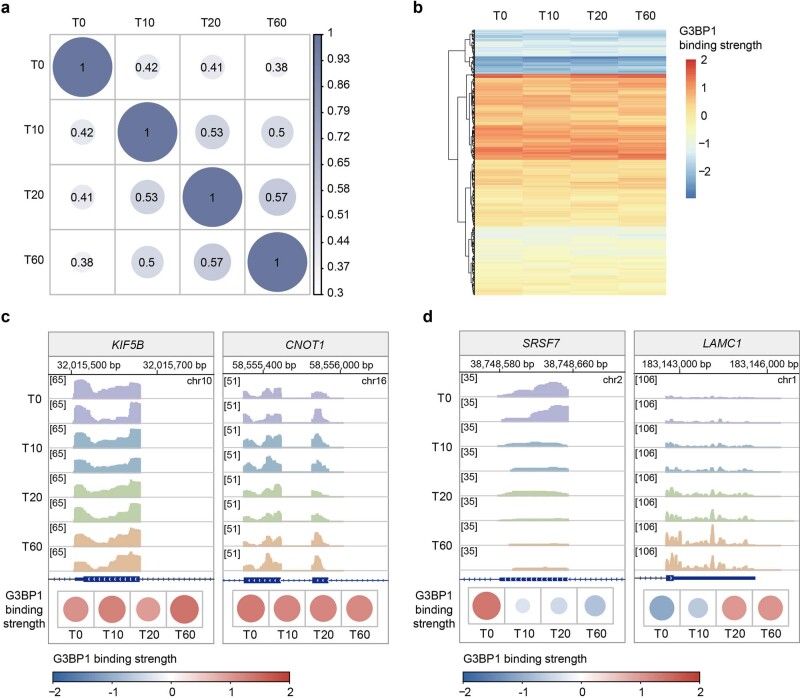
G3BP1 binding at different time intervals during SG assembly captured by ARTR-seq. **a**, The Pearson correlation heatmap among time intervals of ARTR-seq results based on G3BP1 binding strength. G3BP1 binding strength is defined as ARTR-seq read log_2_FC(G3BP1/input). Pairwise correlation coefficients were indicated as circle size and noted in each circle. **b**, A heatmap exhibiting stable G3BP1 binding strength of selected RNAs at different time intervals, organized by hierarchical clustering. RNAs were ranked from small to large according to the standard deviation (SD) of G3BP1 binding intensity over different time intervals, and the top 5% of RNAs were selected for clustering (*n* = 677). The dendrogram was constructed using complete linkage based on Euclidean distance. **c**, IGV snapshots (top) of two RNAs with stable G3BP1 binding strength in ARTR-seq, with each panel normalized by CPM. **d**, IGV snapshots showing RNAs with gradually decreased (left) and increased (right) G3BP1 binding strength. Each panel was normalized by CPM. Heatmaps (bottom) in **c**,**d** show G3BP1 binding strength in ARTR-seq with the size of the circle representing its absolute value.

To further explore the dynamic RNA targeting of G3BP1 over time, we calculated pairwise correlations of the G3BP1 binding strength among time points. The generally low correlation coefficients (*R* = 0.38–0.57) suggested distinct G3BP1 bindings at different time intervals (Extended Data Fig. 10a). RNAs were previously classified into SG-enriched RNAs and SG-depleted RNAs according to their SG enrichment^46^. We found that during SG assembly, G3BP1 binding strength from ARTR-seq gradually increased for SG-enriched RNAs and decreased for SG-depleted RNAs, suggesting a shift of G3BP1 targets toward SG-enriched RNAs (Fig. 6f,g). Some RNAs displayed stable G3BP1 binding, while others showed dynamic G3BP1 binding across time intervals (Fig. 6h and Extended Data Fig. 10b,c). We then grouped these RNAs based on G3BP1 binding strength using the fuzzy *c*-means clustering algorithm. We found that G3BP1 binding strength for these RNAs displayed not only unidirectional trajectories of increasing or decreasing, but also transient changes during 60 minutes of NaAsO_2_ treatment, suggesting rapid and dynamic cellular responses to stress (Fig. 6h,i and Extended Data Fig. 10d). Taken together, ARTR-seq unveiled the highly dynamic nature of G3BP1–RNA interactions during SG assembly, demonstrating its capability in tracking temporal changes of protein–RNA interactions with limited starting materials.

## Discussion

In this work, we present ARTR-seq, a method that captures RBP binding sites using in situ RT by antibody-located RTase. ARTR-seq demonstrated high sensitivity and specificity, even when using as few as 20 cells or limited tissues. The procedure is compatible with immunofluorescence imaging, providing direct spatial information of the targeted proteins without affecting downstream sequencing. With ARTR-seq, we observed the unique binding characteristics of PTBP1, RBFOX2 and HNRNPC related to their splicing regulatory roles. ARTR-seq also detected the preferences of m^6^A reader proteins, YTHDF1, YTHDF2 and YTHDC1. Furthermore, we showed dynamic RNA binding of G3BP1 during SG assembly.

One advantage of ARTR-seq is the use of in situ RT to bypass the antibody-based IP step, thereby reducing material loss. ARTR-seq is also highly versatile and applicable for cell lines, tissues, and even clinical formaldehyde-fixed samples. Both inspired by CUT&Tag^49^, ARTR-seq displays distinct advantages compared to the recently reported RT&Tag^22^. First, ARTR-seq uses random primers to unbiasedly capture local signals, while RT&Tag uses oligo(dT) primer for RT, potentially losing signals from nonpolyadenylated RNAs. Additionally, RT&Tag may experience reduced local resolution due to uniform RT initiation from the poly-A tail and long matured mRNA length (roughly 2,065 bp)^50^, leading to coverage bias toward the RNA 3′ end. Second, Tn5 tagmentation on the RNA–cDNA heteroduplex is less efficient, hindering its applications when using limited starting materials. Third, ARTR-seq can be applied in various cellular compartments, whereas RT&Tag is limited to the isolated nucleus.

Investigations of dynamic RBP binding have been hindered by low UV-crosslinking efficiency, long incubation time and high material demands using the existing methods. Benefiting from highly efficient formaldehyde crosslinking and low starting material requirements, ARTR-seq excels at capturing transient RBP binding across various time intervals. In this work, we have demonstrated its application in capturing dynamic RNA binding of G3BP1 during SG assembly on a timescale of 10 minutes. We envision that the high temporal resolution of ARTR-seq will enable the investigation of dynamic or even transient RBP–RNA interaction in many other events.

### Limitations

The good quality of the primary antibody is a prerequisite for ARTR-seq. For those RBPs without good quality antibodies, ARTR-seq may not accurately capture RBP–RNA interactions. However, the availability of a suitable antibody is a common challenge faced by all antibody-based methods. To overcome this limitation, strategies such as knocking in a tag protein in frame with the targeted RBP or expressing the tagged RBP could be used.

Formaldehyde fixation preserves biological samples at a high temporal resolution, but limitations exist, such as perturbing biomolecular condensates due to the faster protein–protein interaction dynamic than the fixation rate^51^. Strategies to increase the fixation rate, such as increasing the formaldehyde concentration or moderately raising the fixation temperature, can mitigate such artifacts. Like most other methods, ARTR-seq may face challenges when applied to low-abundance RBPs. Approaches such as increasing starting materials or RBP overexpression could be used. Additionally, unlike the editing-based methods, which are compatible with long-read sequencing, ARTR-seq typically shows short fragment lengths (averaging around 60 bp), hindering the identification of isoform-specific binding patterns (Extended Data Fig. 6c). Last, the linker length needs to be optimized when detecting direct versus indirect targets using ARTR-seq, and RNase treatment could be considered to obtain higher resolution binding sites.

## Methods

### Cell culture and stress treatment

HeLa cells (American Type Culture Collection (ATCC) catalog no. CCL-2) and HepG2 cells (ATCC, catalog no. HB-8065) were purchased from ATCC and cultured in DMEM medium (Gibco) supplemented with 10% fetal bovine serum (Gibco) and penicillin-streptomycin (Gibco). K562 cells (ATCC, catalog no. CCL-243) were obtained from ATCC and cultured in RPMI 1640 Medium (Gibco) supplemented with 10% (v/v) fetal bovine serum. Penicillin-streptomycin (Gibco) and 2 mM l-glutamine (Gibco). Cells were grown at 37 °C with 5% CO_2_. For NaAsO_2_ treatment, HeLa cells were grown to 90% confluence and replaced in the prewarmed DMEM medium containing 0.5 mM NaAsO_2_, which was further maintained at 37 °C with 5% CO_2_ for indicated times.

### Expression and purification of recombinant protein A/G-RTase

The recombinant plasmids were constructed by assembly of pet28A vector, protein A/G (pAG), linkers of different lengths and RTase or the modified RTase with NEBuilder HiFi DNA Assembly Master Mix (NEB) or USER enzyme (NEB) following the manufacturer’s protocols. The Protein A/G DNA segment was amplified from the pAG/MNase plasmid (Addgene, catalog no. 123461). The engineered MMLV RTase was modified from the pCMV-PE2 plasmid (Addgene, catalog no. 132775). The recombinant proteins were expressed in BL21(DE3) Competent *Escherichia coli* (NEB) with isopropyl-β-d-thiogalactoside induction at 16 °C for 18 h. Cells were collected by centrifuge at 5,500*g* for 10 min and lysed in the buffer of 50 mM Tris-HCl pH 7.5, 300 mM NaCl and 1 mM PMSF with sonication at 10 s on and 10 s off setting for 10 min at 4 °C. The recombinant proteins were purified from the supernatant using HisTrap HP column (GE Healthcare), followed by an ion exchange chromatography column (GE Healthcare) on an AKTA Purifier 10 system (GE Healthcare) according to the manufacturer’s protocol, and then concentrated to about 20 mg ml^−1^. The purified enzyme was supplemented with 40% glycerol and stored at −80 °C for future use.

### RT–qPCR

RNA was reverse transcribed with the purified pAG-RTases or commercial RTases in reaction buffer (50 mM Tris-HCl, 150 mM NaCl, pH 7.5) at 37 °C for 15 min, and denatured at 85 °C for 5 min. qPCR was performed with FastStart Essential DNA Green Master (Roche) on LightCycler 96 System (Roche). The efficiency of RT was quantified using the delta quantitation cycle method.

### Protein detection by Coomassie brilliant blue stain and western blot

The mammalian cell samples were lysed with cold RIPA buffer (Thermo Fisher Scientific) containing 1× protease inhibitor cocktail (Roche). The cell lysate was cleared with centrifugation at 15,000*g* for 10 min at 4 °C. The supernatant or purified protein was then mixed with LDS loading buffer (Bio-Rad) and boiled at 95 °C for 10 min. Denatured protein was loaded into 4–12% NuPAGE Bis-Tris gel (Thermo Fisher Scientific). For Coomassie brilliant blue stain, the gel was stained with Imperial Protein Stain (Thermo Fisher Scientific) and imaged by FluroChem R (Proteinsimple). For the western blot, the protein was transferred to the polyvinyl difluoride membrane from the gel. The membranes were blocked in 3% BSA (diluted in PBST (PBS with 0.1% Tween-20)) for 1 h at room temperature, incubated in a 1:1,000 diluted primary antibody solution at 4 °C overnight, washed four times with PBST (PBS with 0.1% Tween-20), and incubated in a 1:5,000 dilution of horseradish peroxidase (HRP)-conjugated secondary antibody for 1 h at room temperature if the primary antibody was not conjugated with HRP. The membranes were supplied with SuperSignal West Dura Extended Duration Substrate kit (Thermo Fisher Scientific) and imaged on the FluroChem R machine (Proteinsimple). Quantification was performed using ImageJ software (v.2.3.0).

### Transfection

PTBP1 siRNA was purchased from Horizon Discovery/Dharmacon. Cells were seeded in 30% confluency. After incubation for 12 h, siRNA was transfected with RNAimax (Thermo Fisher Scientific) following the manufacturer’s manual. The fresh medium was changed at 6 h posttransfection. Cells were cultured for another 48 h, and the protein knockdown efficiency was quantified by western blot.

### ARTR-seq

Cells were fixed to an imaging-compatible chamber with 1.5% paraformaldehyde (PFA) at room temperature for 10 min. To mitigate cell loss, 1.5% PFA crosslinking was applied instead of the commonly used 1% PFA crosslinking. The samples were then quenched with 125 mM glycine at room temperature for 5 min, washed twice with Dulbecco’s PBS (DPBS) and permeabilized with 0.5% Triton X-100 in DPBS on ice for 10 min. Each DPBS washing step involved 3 min of incubation at room temperature. Next, samples were washed twice with DPBS, blocked with the blocking buffer (1 mg ml^−1^ UltraPure BSA, 0.2 U μl^−1^ RNaseOUT in DPBS) at room temperature for 30 min and stained with the diluted primary antibody at room temperature for 1 h. The primary antibody was diluted with blocking buffer according to the manufacturer’s instructions for immunofluorescence or at a 1:200 dilution if no specific guidance was provided. For input samples, the primary antibody diluent was replaced by the blocking buffer. Subsequently, samples were stained with fluorophore-labeled secondary antibody (1:500 diluted in the blocking buffer) at room temperature for 30 min, followed by incubation with pAG-RTase (10 nM in the blocking buffer) for an additional 30 min. Cells were washed three times with DPBS after each staining step by shaking at room temperature for 3 min.

An RT reaction mixture was prepared by mixing 2 μM adapter-RT primer (5′-AGACGTGTGCTCTTCCGATCTNNNNNNNNNN-3′), 0.05 mM biotin-16-dUTP (Jena Bioscience), 0.05 mM biotin-16-dCTP (Jena Bioscience), 0.05 mM dTTP (Thermo Fisher Scientific), 0.05 mM dCTP (Thermo Fisher Scientific), 0.1 mM dATP (Thermo Fisher Scientific), 0.1 mM dGTP (Thermo Fisher Scientific), 1 U μl^−1^ RNaseOUT (Thermo Fisher Scientific) in 50 μl buffer of DPBS supplemented with 3 mM MgCl_2_. In situ RT was performed by immersing cells with the RT reaction mixture and incubating at 37 °C for 30 min, then stopped by adding 20 mM EDTA and 10 mM EGTA and incubating at room temperature for 3 min.

Next, cells were stained with 1:200 diluted biotin monoclonal antibody (BK-1/39), alexa fluor 488 (Thermo Fisher Scientific) in DPBS by incubation at room temperature for 1 h, followed by staining with 1 μg ml^−1^ Hoechst 33342 dye (Thermo Fisher Scientific) at room temperature for 15 min. The samples were then imaged by Leica SP8 laser confocal microscope. The fluorescence intensity distribution on a line was quantified by ImageJ software.

After imaging, cells were digested with 1 mg ml^−1^ proteinase K (Thermo Fisher Scientific) at 37 °C for 2 h. The nucleic acids were recovered by phenol-chloroform extraction (pH 8.0) and concentrated by ethanol precipitation. RNA was digested with 0.2 U μl RNase H (NEB) and 1:20 diluted RNase A/T1 (Thermo Fisher Scientific) in 50 μl of the RNase reaction buffer (50 mM Tris-HCl pH 7.5, 75 mM KCl, 10 mM MgCl_2_, 10 mM DTT) at 37 °C for 1 h, followed by biotinylated cDNA enrichment using 10 μl preblocked Dynabeads MyOne Streptavidin C1 (Thermo Fisher Scientific) at room temperature for 20 min. The beads were preblocked with 1 μg μl^−1^ UltraPure BSA (Thermo Fisher Scientific), 1 μg μl^−1^ UltraPure Salmon Sperm DNA Solution (Thermo Fisher Scientific) and 1 μg μl^−1^ Yeast transfer RNA (tRNA) (Thermo Fisher Scientific) with incubation at room temperature for 30 min before performing biotinylated cDNA enrichment.

Subsequently, the cDNA adapter ligation mixture was prepared by combining 50 mM Tris-HCl pH 7.5, 10 mM MgCl_2_, 25% PEG 8000, 1 mM ATP, 1 U μl^−1^ T4 RNA ligase 1 (NEB), and 5 μM of 3′ cDNA adapter (5′Phos-NNNNNNNNAGATCGGAAGAGCGTCGTGT-3′SpC3). The 3′ cDNA adapter ligation was performed by suspending the beads in the cDNA adapter ligation mixture and incubating at 25 °C for 16 h. The biotinylated cDNA was recovered using an elution buffer composed of 95% (v/v) formamide and 10 mM EDTA (pH 8.0) by boiling at 95 °C for 10 min, followed by ethanol precipitation. The cDNA was then dissolved in 10 μl of water.

For library amplification, 40 μl of mixture was prepared by mixing 1× NEBNext Ultra II Q5 Master Mix (NEB), 10 μl of cDNA solution and 0.5 μM Illumina sequencing primers, such as NEBNext Multiplex Oligos for Illumina (NEB catalog no. E7335S). The library PCR amplification followed this program: 98 °C for 30 s (98 °C for 10 s, 60 °C for 30 s, 65 °C for 45 s) for 13 cycles and 65 °C for 5 min; hold at 4 °C. The final libraries were purified using 6% Novex TBE Gel (Thermo Fisher Scientific) with size selection between 180 and 400 bp. Next-generation sequencing was carried out either at the University of Chicago Single Cell Immunophenotyping Core on an Illumina NextSeq 550 machine or Illumina NextSeq 2000 machine, or at the University of Chicago Genomics Facility on an Illumina NovaSeq 6000 platform.

### RNase treatment in ARTR-seq

RNase treatment was incorporated into the ARTR-seq procedure with the following modifications: After permeabilization, Cells were incubated with 1 U μl^−1^ RNase I (Thermo Fisher Scientific) at 37 °C for 5 min, followed by two washes with DPBS. For samples with strong RNase treatment, an additional RNase I treatment was conducted as previously described before initiating RT.

### Dot blot

After the proteinase K digestion step in ARTR-seq, the total nucleic acids were recovered with Oligo Clean & Concentrator Kits (Zymo) to get rid of free biotinylated dNTP. The concentration of nucleic acids was measured by Nanodrop 8000 Spectrophotometer and adjusted to 50 ng µl^−1^. Next, 1 µl of nucleic acids were loaded onto the Amersham Hybond- N+ membrane (GE Healthcare). Membranes were air-dried and crosslinked by UV strata linker 2400 at 150 mJ cm^−2^ twice. The membranes were then blocked in 5% fatty-acid-free BSA in PBST at room temperature for 1 h, followed by incubation in streptavidin-HRP (Thermo Fisher Scientific) in PBST supplemented with 5% fatty-acid free BSA at room temperature for another 1 h. The membrane was washed with PBST four times before being supplied with SuperSignal West Dura Extended Duration Substrate kit (Thermo Fisher Scientific) and imaged by the FluroChem R machine (Proteinsimple).

### ARTR-seq in the mouse embryo

C57 mouse embryo (E11) frozen tissue sections were purchased from Zyagen. The slide with frozen tissue sections was brought to room temperature for 10 min of incubation. The PAP pen was used to draw a circle around the mouse tissue on the slide, providing a thin film-like hydrophobic barrier for reagent incubation. Then the tissue was subjected to typical ARTR-seq procedures with the following change. The 2 μM adapter-barcoded RT primer (5′-AGACGTGTGCTCTTCCGATCT-(8 nt-barcode)-NNNNNNNNNN-3′) was applied for in situ RT.

### ARTR-seq with low input

ARTR-seq was applied to 20 to 5,000 HepG2 cells with the following changes. 4% PFA was used to minimize cell loss for low-input samples. The 2 μM adapter-barcoded RT primer (5′-AGACGTGTGCTCTTCCGATCT-(8 nt-barcode)-NNNNNNNNNN-3′) was applied for in situ RT. After digestion of proteinase K, two biological replicates were pooled together for biotinylated cDNA enrichment, adapter ligation, library amplification and library sequencing. Sequence data were isolated based on the 8 nt barcode in adapter-barcoded RT primers.

### Genome reference

Genome and the corresponding reference of *Homo sapiens* (GRCh38.p13, GENCODE Release 39), *Mus musculus* (GRCm39, GENCODE Release M29) and *Drosophila melanogaster* (BDGP6.32, Ensembl Release 107) were used for mapping the sequencing reads in this study. Ribosomal RNA (rRNA) reference sequences were downloaded from the National Center for Biotechnology Information (NCBI) for *H. sapiens* (NR_003285.3, NR_003286.4, NR_003287.4, NR_023363.1), *M. musculus* (NR_003278.3, NR_003279.1, NR_003280.2, NR_046156.1) and from FlyBase for *D. melanogaster* (5SrRNA-CR33353, 18SrRNA-CR45841, 5.8SrRNA-CR45842 and 28SrRNA-CR4584)

### ARTR-seq primary data processing

Reads from the small cell number libraries containing cell barcodes were first demultiplexed with an in-house script using read 2. The adapter sequences were trimmed with Cutadapt^54^ (v.4.2) using the parameter cutadapt–nextseq-trim=20 -a AGATCGGAAGAGCACACGTCTGAACTCCAG; the 8 nt unique molecular identifier sequences were moved and add to the read name for the further deduplication. An extra 4 nts at the reads’ 3′ end were removed from the adapter-free sequence to minimize mapping mismatch caused by the imperfect paired sequence in the random primer.

The reads were first mapped to the corresponding rRNA sequences using Bowtie2 (ref. ^55^) (v.2.4.4) with parameters: –seedlen=15, and the mapped reads were discarded to avoid rRNA contamination. The remaining unmapped reads were mapped to the corresponding genome using STAR^56^ (v.2.7.9a) with parameters: –readFilesCommand zcat–alignEndsType EndToEnd–genomeLoad NoSharedMemory–quantMode TranscriptomeSAM–alignMatesGapMax 15000–outFilterMultimapNmax 1–outFilterMultimapScoreRange 1–outSAMprimaryFlag AllBestScore–outSAMattributes All–outSAMtype BAM SortedByCoordinate–outFilterType BySJout–outReadsUnmapped Fastx–outFilterScoreMin 10–outFilterMatchNmin 24. Uniquely mapped reads were deduplicated to get the usable reads using UMI-tools^57^ (v.1.1.2) with the parameter, –method unique. The usable reads were assigned to genomic regions with RNASeQC^58^ (v.2.4.2) using default parameters. Deduplicated reads were assigned to genes with featureCounts^59^ (v.2.0.3) for the calculation of Pearson’s correlation coefficient between biological replicates. For visualization in IGV^60^ (v.2.13.1), .bam files of the usable reads were converted to bigWig with bamCoverage in the deepTools suite^61^ (v.3.5.1) with normalization by its respective sequencing depth using the parameters –normalizeUsing BPM–binSize 1. All the sample tracks were set to the same scale for display, except for the additional instruction noted in the legend.

### Peaking calling

For peak calling, we first split the usable reads in one library into two .bam files containing reads aligned to the positive and negative strands, respectively. We used macs3 (ref. ^62^) to identify peaks with default parameters, except for adding ‘–keep-dup all–nomodel –extsize 30’. macs3 gives the fold enrichment (signal value) and *P* value based on Poisson distribution, and corrects the *P* values for multiple comparison using the Benjamini–Hochberg correction. The peaks located in two strands were called separately using the corresponding strand read in the input libraries as background. The two peak files for one library were later combined. To generate the consensus motif for peaks, we first extended 20 nts to both upstream and downstream, and the overrepresented sequences were generated using findMotifsGenome.pl in the HOMER suite^52^ (v.4.11) with parameters: -rna -S 10 -len 5,6,7,8,9. Specifically, for motif generation for peaks in mouse tissue, the peak genomic coordinates were converted from mm39 to mm10 using liftOver from the UCSC Genome Browser^63^. Peaks were assigned to specific genomic regions with in-house scripts, and the peaks overlapping two genomic regions were assigned to the region of longer overlapping base pairs. The peaks from the reader YTHDC1 were further assigned to repeats and other regions with annotatePeaks.pl in the HOMER suite.

### Subsampling

To calculate the percentage of usable reads at different sequencing depths, we subsample the uniquely mapped reads with the samtools view in the Samtools suite^64^ (v.1.16.1). For the comparison between small cell number input libraries for different methods, the sizes of all libraries were reduced to that of the smallest library. Specifically, instead of directly subsampling the fastq files, we subsampled the uniquely mapped reads to calculate the usable read percentage of each library.

### Alternative splicing identification

The differential alternative splicing events of each gene were identified using rMATS (v.4.1.2). The RBP-knockdown RNA-seq libraries bam files and the corresponding control libraries’ .bam files with the annotation of ENCODE4 v.1.2.1 GRCh38 V29 were downloaded from the ENCODE and were analyzed by rMATS for the identification of five alternative splicing modes, including skipped exon, mutually exclusive exons, alternative 3′ splice site, alternative 5′ splice site and retained introns. Events of FDR ≥ 0.05 were discarded for the subsequent analysis.

### ARTR-seq enrichment level at the gene level

To calculate the ARTR-seq enrichment at the gene level, we divided the reads in one library into two groups by whether they were in one specific gene, and had a pair of in–out read numbers for each of the IP and Input libraries. For each gene, we generated two-by-two tables for all the combinations of in–out read numbers between IP and Input libraries. The ARTR-seq enrichment for a gene is defined as the common odds ratio of the tables with significance determined by the Cochran–Mantel–Haenszel chi-squared test.

### Data visualization and statistical analysis

Read heatmaps and profiles were generated with plotHeatmap and plotProfile in the deepTools suite^61^ (v.3.5.1), using genomic coordinates unless otherwise indicated. The splicing maps of splicing factors are generated by RBP-Maps^37^ with default parameters in the ‘Plotting peaks’ mode (–peak), and the hg19 coordinates of native cassette exons and constitutive exons were downloaded from the software GitHub deposit. The peak genomic coordinates of the peaks for the splicing factors were first converted from GRCh38 to hg19 using liftOver from the UCSC Genome Browser^63^. The random regions are random exonic regions with the same length as pooled ARTR-seq peaks from the three m^6^A reader proteins, generated by bedtools shuffle in the BEDTools suite^65^ (v.2.30.0).

The meta distributions of binding peaks were generated by the R package Guitar^66^ (v.2.16.0). All statistical analyses were performed with R^67^, and all the plots were generated by the R package ggplot2 (ref. ^68^) (v.3.4.1).

### Quantification of ARTR-seq signal at the gene level

To analyze G3BP1 binding strength at the gene level, ARTR-seq reads were counted for genes in both G3BP1 and paired input samples, and FCs and significance between G3BP1 and input were determined by DESeq2 (ref. ^69^). Only genes with the read sum equal to or greater than ten for G3BP1 and input samples were considered. RNA targets of G3BP1 were defined as those with a FC ≥ 2 and *P* < 0.05. Both FC and *P* value were calculated by DESeq2 with the default setting.

### Clustering analysis of G3BP1 ARTR-seq signal

To track the changing pattern of G3BP1 binding single during the SG assembly, we used log_2_FC (G3BP1/input) of genes to represent the G3BP1 binding signal, and performed fuzzy *c*-means clustering analysis on log_2_FC by the Mfuzz package^70^ (v.2.54.0). Only genes with the top 50% of the greatest standard deviation (s.d.) of log_2_FC were considered, and the log_2_FC values were scaled by *z* score before clustering. The cluster number was determined by the ‘Dmin’ function in the Mfuzz package. Clustering was calculated by the ‘mfuzz’ function in the Mfuzz package with 10,000 iterations with Euclidean distance as the clustering method. The membership values indicate the degree of association of genes with their respective clusters.

### Functional enrichment analysis

KEGG enrichment analysis was carried out to compare G3BP1–RNA targets at different time points using the ‘compareCluster’ function in the clusterProfiler package^53^ (v.4.4.4). The KEGG terms with adjusted *P* values less than 0.05 were visualized.

### Statistics and reproducibility

Unless otherwise stated, a two-tailed Student’s *t*-test or Wilcoxon test were performed to assess the statistical significance between groups. The resulting *P* values are indicated in the figure or legends. For boxplots, the box represents the 25th to 75th percentiles with a line at the median, whiskers to 1.5 times the interquartile range, a dot at the mean (if applicable) and outliers omitted. Immunofluorescence imaging experiments were repeated in at least two biological samples with consistent results.

### Reporting summary

Further information on research design is available in the Nature Portfolio Reporting Summary linked to this article.

## Online content

Any methods, additional references, Nature Portfolio reporting summaries, source data, extended data, supplementary information, acknowledgements, peer review information; details of author contributions and competing interests; and statements of data and code availability are available at 10.1038/s41592-023-02146-w.

### Supplementary information

Supplementary Information Supplementary Fig. 1 and Tables 1–3.

Reporting Summary

### Source data

Source Data Fig. 1 Unprocessed dot blot.

Source Data Fig. 1 Statistical source data.

Source Data Extended Data Fig. 1 Unprocessed gel.

Source Data Extended Data Fig. 1 Statistical source data.

Source Data Extended Data Fig. 3 Unprocessed western blots.

Source Data Extended Data Fig. 3 Statistical source data.

Source Data Extended Data Fig. 5 Unprocessed gel.

Source Data Extended Data Fig. 5 Statistical source data.

Source Data Extended Data Fig. 6 Statistical source data.

## Data Availability

All the sequencing data generated in this study have been deposited in the NCBI’s Gene Expression Omnibus (GEO) under the accession number GSE226161. Previously published data are available under accession numbers GSE42701 (CLIP-seq^26^), ENCSR384KAN and ENCSR981WKN (eCLIP^28^), E-MTAB-3108 (iCLIP^27^), GSE78832 (irCLIP^10^), GSE137925 (LACE-seq^13^), GSE92995 (sCLIP^11^), DRA005743 (tRIP-seq^12^) and GSE195654 (RT&Tag^22^). The data were downloaded and processed as described in the articles. The processed .bam files of RNA-seq data for knockdown HNRNPC, PTBP1 and RBFOX2, along with their corresponding control data, were downloaded from ENCODE portal^28^ under the accession numbers of ENCSR052IYH, ENCSR305XWT, ENCSR767LLP, ENCSR104ABF, ENCSR064DXG and ENCSR603TCV. The published PAR-CLIP data and the corresponding peaks for YTHDF2 are available under the GEO accession number GSE49339. The m^6^A modification sites identified by m^6^A-SAC-seq are available under the GEO accession number GSE198246. Source data are provided with this paper.

## Code availability

Codes for processing ARTR-seq data are available in the following GitHub repository: https://github.com/mingming-cgz/ARTR-seq.
