# Supplementary material for: Profiling of RNA-binding protein binding sites by in situ reverse transcription-based sequencing
Source: Nat Methods. 2024 Jan 10;21(2):247–58. doi: 10.1038/s41592-023-02146-w (PMC10864177; doi:10.1038/s41592-023-02146-w)
Supplement: Supplementary file 1 — Supplementary Fig. 1 and Tables 1–3. [file 41592_2023_2146_MOESM1_ESM.pdf]

nature methods

Article

https://doi.org/10.1038/s41592-023-02146-w

Profiling of RNA-binding protein binding sites by in situ reverse transcription-based sequencing

In the format provided by the authors and unedited

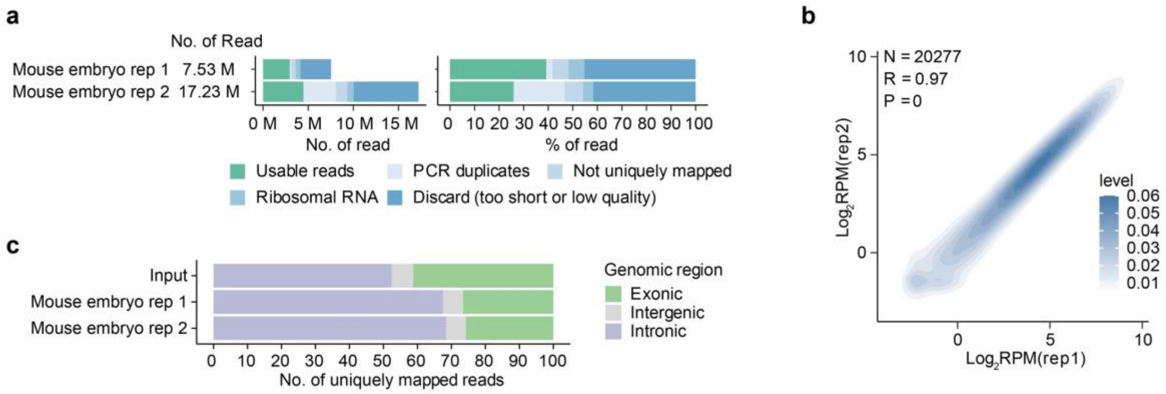

Supplementary Fig. 1. Quality of ARTR-seq libraries using mouse embryo tissues.
a, Bar plots showing numbers (left) and percentages (right) of the usable reads and reads filtered after each processing step for ARTR-seq libraries. **b**, ARTR-seq replicate correlations for usable reads per gene normalized to coverage (RPM) for RBFOX2 in mouse embryos. The color scale shows the point distribution density. The coefficient R and P-values were given by the two-tailed Pearson's correlation. **c**, A bar plot showing the usable reads distribution in the intronic (purple), intergenic (grey) and exonic (green) regions of ARTR-seq libraries constructed from mouse embryos.

Gene Name	Gene ID	Primer direction	Primer sequence (5′ to 3′)
GAPDH	2597	Forward	GTCTCCTCTGACTTCAACAGCG
GAPDH	2597	Reverse	ACCACCCTGTTGCTGTAGCCAA
ACTB	60	Forward	CACCATTGGCAATGAGCGGTTC
ACTB	60	Reverse	AGGTCTTTGCGGATGTCCACGT
METTL3	56339	Forward	CTATCTCCTGGCACTCGCAAGA
METTL3	56339	Reverse	GCTTGAACCGTGCAACCACATC
METTL14	57721	Forward	CTGAAAGTGCCGACAGCATTGG
METTL14	57721	Reverse	CTCTCCTTCATCCAGATACTTACG
RBM15	29890	Forward	TGGTAACCTGGACCACAGCGTA
RBM15	29890	Reverse	GGTTCTGGAACTTGAGGAAGGC

Supplementary Table 1. Quantitative PCR primers.

Name	Manufactory	Target sequence (5′ to 3′)
PTBP1 siRNA-1	Horizon Discovery	GGCACAAGCUGCACGGGAA
PTBP1 siRNA-2	Horizon Discovery	GCAUCACGCUCUCGAAGCA

Supplementary Table 2. siRNA sequence for *PTBP1* knockdown.

Method	Cell number	Cell line	Type	Sample size (number of usable reads)
ARTR	40 k	HepG2	Input	4596513
ARTR	40 k	HepG2	PTBP1	9518232
ARTR	40 k	HepG2	PTBP1	7664034
ARTR	5 k	HepG2	PTBP1	3285354
ARTR	2 k	HepG2	PTBP1	4751687
ARTR	1 k	HepG2	PTBP1	1947829
ARTR	500	HepG2	PTBP1	2962287
ARTR	200	HepG2	PTBP1	1947023
ARTR	100	HepG2	PTBP1	1576131
ARTR	50	HepG2	PTBP1	1173292
ARTR	20	HepG2	PTBP1	465677
CLIP	20 M	HeLa	PTBP1	12771193
iCLIP	10 M	HeLa S3	PTBP1	1752858

iCLIP	10 M	HeLa S3	PTBP1	3168007
eCLIP	20 M	HepG2	Input	6171975
eCLIP	20 M	HepG2	PTBP1	7323798
eCLIP	20 M	HepG2	PTBP1	4422769
irCLIP	1.8 M	HeLa	PTBP1	34315701
irCLIP	1.8 M	HeLa	PTBP1	27115619
LACE	100 k	HeLa	PTBP1	12986474
LACE	100 k	HeLa	PTBP1	15265703
LACE	100 k	K562	PTBP1	2771113
LACE	100 k	K562	PTBP1	3940355

The arrangement of the elements in the table (from top to bottom) corresponds to the box layout in **Figure 2h** (from left to right).

Supplementary Table 3. Sample size for Figure 2h.
