# Supplementary material for: Profiling of RNA-binding protein binding sites by in situ reverse transcription-based sequencing
Source: Nat Methods. 2024 Jan 10;21(2):247–58. doi: 10.1038/s41592-023-02146-w (PMC10864177; doi:10.1038/s41592-023-02146-w)
Supplement: Supplementary file 2 — Reporting Summary [file 41592_2023_2146_MOESM2_ESM.pdf]

nature portfolio

Corresponding author(s): Chuan He

Last updated by author(s): Nov 5, 2023

Reporting Summary

Nature Portfolio wishes to improve the reproducibility of the work that we publish. This form provides structure for consistency and transparency in reporting. For further information on Nature Portfolio policies, see our Editorial Policies and the Editorial Policy Checklist.

Statistics

For all statistical analyses, confirm that the following items are present in the figure legend, table legend, main text, or Methods section.

n/a	Confirmed	
☐	☒	The exact sample size (*n*) for each experimental group/condition, given as a discrete number and unit of measurement
☐	☒	A statement on whether measurements were taken from distinct samples or whether the same sample was measured repeatedly
☐	☒	The statistical test(s) used AND whether they are one- or two-sided<br>*Only common tests should be described solely by name; describe more complex techniques in the Methods section.*
☒	☐	A description of all covariates tested
☒	☐	A description of any assumptions or corrections, such as tests of normality and adjustment for multiple comparisons
☐	☒	A full description of the statistical parameters including central tendency (e.g. means) or other basic estimates (e.g. regression coefficient) AND variation (e.g. standard deviation) or associated estimates of uncertainty (e.g. confidence intervals)
☐	☒	For null hypothesis testing, the test statistic (e.g. *F*, *t*, *r*) with confidence intervals, effect sizes, degrees of freedom and *P* value noted<br>*Give P values as exact values whenever suitable.*
☒	☐	For Bayesian analysis, information on the choice of priors and Markov chain Monte Carlo settings
☒	☐	For hierarchical and complex designs, identification of the appropriate level for tests and full reporting of outcomes
☐	☒	Estimates of effect sizes (e.g. Cohen's *d*, Pearson's *r*), indicating how they were calculated

Our web collection on statistics for biologists contains articles on many of the points above.

Software and code

Policy information about availability of computer code

Data collection	Illuminal NextSeq 550 System, Illumina NovaSeq 6000 System, Illuminal NextSeq 2000 System
Data analysis	Cutadapt (v4.2), Bowtie2 (v2.4.4), STAR (v2.7.9a), UMI-tools (v1.1.2), RNASeQC (v2.4.2), featureCounts (v2.0.3), IGV (v2.13.1), deepTools (v3.5.1), HOMER (v4.11), UCSC Genome Browse LiftOver (no version no. shown in the website, downloaded on Aug. 4th , 2021), Samtools (v1.16.1), rMATS (v4.1.2), RBP-Maps (v0.1.4), BEDTools (v2.30.0), R (v4.2), Guitar (v2.16.0), Mfuzz (v2.54.0), ggplot2 (v 3.4.1), clusterProfiler (v4.4.4), ImageJ (v2.3.0)<br>Codes for processing ARTR-seq data are available in the following GitHub repository https://github.com/mingming-cgz/ARTR-seq.

For manuscripts utilizing custom algorithms or software that are central to the research but not yet described in published literature, software must be made available to editors and reviewers. We strongly encourage code deposition in a community repository (e.g. GitHub). See the Nature Portfolio guidelines for submitting code & software for further information.

Data

Policy information about availability of data

All manuscripts must include a data availability statement. This statement should provide the following information, where applicable:

- Accession codes, unique identifiers, or web links for publicly available datasets
- A description of any restrictions on data availability
- For clinical datasets or third party data, please ensure that the statement adheres to our policy

All the sequencing data generated in this study have been deposited in NCBI's Gene Expression Omnibus (GEO) under the accession number GSE226161. Previously published data from CLIP-seq, eCLIP, iCLIP, irCLIP, LACE-seq, sCLIP, tRIP-seq and RT&Tag are available under accession numbers of GSE42701, GSE92205, E-MTAB-3108, GSE78832, GSE137925, GSE92995, DRA005743 and GSE195654, respectively. The data were downloaded and processed as described in those articles. The PTBP1, RBFOX2 and HNRNPC knockdown RNA-Seq data were downloaded from ENCODE portal under the accession numbers of ENCSR052IYH, ENCSR305XWT, ENCSR634KBO, ENCSR572FFX, ENCSR767LLP, ENCSR104ABF, ENCSR336DFS, ENCSR667PLJ, ENCSR064DXG, ENCSR603TCV, ENCSR527IVX, ENCSR129RWD. The published PAR-CLIP data and the corresponding peaks for YTHDF2 are available under the GEO accession number of GSE49339. The m6A modification sites list identified by m6A-SAC-seq is available under the GEO accession number of GSE198246.

Genome and the corresponding reference of Homo sapiens (GRCh38.p13, GENCODE Release 39), Mus musculus (GRCm39, GENCODE Release M29), and Drosophila melanogaster (BDGP6.32, Ensembl Release 107) were used for mapping the sequencing reads in this study. rRNA reference sequences were downloaded from NCBI for H. sapiens (NR_003285.3, NR_003286.4, NR_003287.4, NR_023363.1), M. musculus (NR_003278.3, NR_003279.1, NR_003280.2, NR_046156.1), and from FlyBase for D. melanogaster (5SrRNA-CR33353, 18SrRNA-CR45841, 5.8SrRNA-CR45842, 28SrRNA-CR4584)

Human research participants

Policy information about studies involving human research participants and Sex and Gender in Research.

Reporting on sex and gender	N/A. This work does not involve human research participants.
Population characteristics	N/A. This work does not involve human research participants.
Recruitment	N/A. This work does not involve human research participants.
Ethics oversight	N/A. This work does not involve human research participants.

Note that full information on the approval of the study protocol must also be provided in the manuscript.

Field-specific reporting

Please select the one below that is the best fit for your research. If you are not sure, read the appropriate sections before making your selection.

☒ Life sciences ☐ Behavioural & social sciences ☐ Ecological, evolutionary & environmental sciences

For a reference copy of the document with all sections, see nature.com/documents/nr-reporting-summary-flat.pdf

Life sciences study design

All studies must disclose on these points even when the disclosure is negative.

Sample size	No statistical methods were used to predetermine sample size. All sample sizes were determined based on our prior experience on similar experiments and literature reports (Wu et al., Nat Meth, 2020, PMID: 32251394; Su et al., Nat Cell Biol, 2021, PMID: 34108658)
Data exclusions	The adaptor sequences were trimmed; the 8-nt UMI sequences were moved and add to the read name for the further deduplication. Extra 4 nt at the reads' 3-end were removed from the adapter-free sequence to minimize mapping mismatch caused by the imperfect paired sequence in the random primer. The corresponding rRNA were discarded to avoid rRNA contamination.
Replication	Results were confirmed in two biological replicates for each experiment unless otherwise stated. All attempts to replicate data are successful.
Randomization	The experiments were not randomized. Controlling for covariates was unnecessary because all assays were performed in pairs.
Blinding	Blinding was not applicable because the focus of this paper is the development of a new method to capture RNA-binding protein binding sites and did not involve group allocation and blinding.

Reporting for specific materials, systems and methods

We require information from authors about some types of materials, experimental systems and methods used in many studies. Here, indicate whether each material, system or method listed is relevant to your study. If you are not sure if a list item applies to your research, read the appropriate section before selecting a response.

Materials & experimental systems

n/a	Involved in the study
☐	☒ Antibodies
☐	☒ Eukaryotic cell lines
☒	☐ Palaeontology and archaeology
☐	☒ Animals and other organisms
☒	☐ Clinical data
☒	☐ Dual use research of concern

Methods

n/a	Involved in the study
☒	☐ ChIP-seq
☒	☐ Flow cytometry
☒	☐ MRI-based neuroimaging

Antibodies

Antibodies used

PTBP1 (Cell Signaling Technology, Cat. No. 57246, clone No. E4I3Q)
RBFOX2 (Proteintech, Cat. No. 12498-1-AP)
HNRNPC (Santa Cruz, Cat. No. sc-32308, clone No. 4F4)
YTHDF1 (abcam, Cat. No. ab220162, clone No. EPR22349-41)
YTHDF2 (abcam, Cat. No. ab220163, clone No. EPR20318)
YTHDC1 (abcam, Cat. No, ab122340)
G3BP1 (BD Biosciences, Cat. No. 611126, clone No. 23/G3BP (RUO))
Biotin monoclonal antibody - alexa fluor 488 (Thermo Fisher Scientific, Cat. No. 53-9895-82, clone No. BK-1/39)
Rabbit anti-GAPDH mAb-HRP Conjugate (Cell Signaling Technology, Cat. No. 3683, clone No. 14C10)
Goat Anti-Rabbit IgG (H+L)-HRP Conjuate (Cell Signaling Technology, Cat. No. 7074)
Goat anti-Rabbit IgG (H+L) Highly Cross-Adsorbed Secondary Antibody, Alexa Fluor™ 568 (Thermo Fisher Scientific, Cat. No. A-11036)
Goat anti-Mouse IgG (H+L) Highly Cross-Adsorbed Secondary Antibody, Alexa Fluor™ 568 (Thermo Fisher Scientific, Cat. No. A-11031)

Validation

PTBP1: IF validated in our hand. Manufactures: Rabbit monoclonal, applications in WB, IP; Validated species: Human, Mouse, Rat, Monkey (https://www.cellsignal.com/products/primary-antibodies/ptbp1-e4i3q-rabbit-mab/57246)
RBFOX2: IF validated in our hand. Manufactures: knockout validated, Rabbit polyclonal, applications in WB, IP, IHC, IF, ELISA; Validated species: Human, Mouse (https://ptglab.com/products/RBM9-Antibody-12498-1-AP.htm)
HNRNPC: IF validated in our hand. Manufactures: Mouse monoclonal, applications in WB, IP, IHC, FITC, IF; Validated species: Human (https://www.scbt.com/p/hnrnp-c1-c2-antibody-4f4?bvstate=pg:2/ct:r)
YTHDF1: IF validated in our hand. Manufactures: knockout validated, Rabbit monoclonal, applications in WB, IP; Validated species: Mouse, Rat, Human (https://www.abcam.com/products/primary-antibodies/ythdf1-antibody-epr22349-41-ab220162.html)
YTHDF2: IF validated in our hand. Manufactures: knockout validated, Rabbit monoclonal, applications in WB, IP; Validated species: Mouse, Rat, Human (https://www.abcam.com/products/primary-antibodies/ythdf2-antibody-epr20318-ab220163.html)
YTHDC1: IF validated in our hand. Manufactures: Rabbit polyclonal, applications in ICC/IF, IHC-P, WB; Validated species: Human (https://www.abcam.com/products/primary-antibodies/ythdc1-antibody-ab122340.html)
G3BP1: IF validated in our hand. Manufactures: Mouse monoclonal, applications in WB, IF; Validated species: Human(https://www.bdbiosciences.com/en-us/products/reagents/microscopy-imaging-reagents/immunofluorescence-reagents/purified-mouse-anti-human-g3bp.611126)
Biotin monoclonal antibody - alexa fluor 488: IF validated in our hand. Manufactures: Mouse monoclonal, applications in WB, FACS (https://www.thermofisher.com/antibody/product/Biotin-Antibody-clone-BK-1-39-Monoclonal/53-9895-82)
Rabbit anti-GAPDH mAb-HRP Conjugate: WB validated in our hand. Manufactures: monoclonal, applications in WB; Validated species: Human, Mouse, Rat, Monkey, Bovine, Pig (https://www.cellsignal.com/products/antibody-conjugates/gapdh-14c10-rabbit-mab-hrp-conjugate/3683)
Goat Anti-Rabbit IgG (H+L)-HRP Conjuate: WB validated in our hand. Manufactures: applications in WB; Validated species: Rabbit (https://www.cellsignal.com/products/secondary-antibodies/anti-rabbit-igg-hrp-linked-antibody/7074)
Goat anti-Rabbit IgG (H+L) Highly Cross-Adsorbed Secondary Antibody, Alexa Fluor™ 568: IF validated in our hand. Manufactures: Goat ponoclonal, applications in IF, ICC, IHC; Validated species: Rabbit (https://www.thermofisher.com/antibody/product/Goat-anti-Rabbit-IgG-H-L-Highly-Cross-Adsorbed-Secondary-Antibody-Polyclonal/A-11036)
Goat anti-Mouse IgG (H+L) Highly Cross-Adsorbed Secondary Antibody, Alexa Fluor™ 568: IF validated in our hand. Manufactures: Goat ponoclonal, applications in IF, ICC, FACS; Validated species: Mouse (https://www.thermofisher.com/antibody/product/Goat-anti-Mouse-IgG-H-L-Highly-Cross-Adsorbed-Secondary-Antibody-Polyclonal/A-11031)

Eukaryotic cell lines

Policy information about cell lines and Sex and Gender in Research

Cell line source(s)

HepG2 cells are purchased from ATCC (catalog No: HB-8065).
HeLa cells are purchased from ATCC (catalog No: CCL-2).
K562 cells are purchased from ATCC (catalog No: CCL-243).

Authentication

Cell lines were not authenticated after purchase from ATCC.

Mycoplasma contamination

All cell lines used in this study were tested negative of mycoplasma contamination.

March 2021

Commonly misidentified lines (See ICLAC register)	No commonly misidentified line was used.

Animals and other research organisms

Policy information about studies involving animals; ARRIVE guidelines recommended for reporting animal research, and Sex and Gender in Research

Laboratory animals	This study did not involve laboratory animals. The C57 mouse embryo (E11) frozen tissue sections were purchased from Zyagen.
Wild animals	This study did not involve wild animals.
Reporting on sex	This study did not involve sex.
Field-collected samples	This study did not involve samples collected from field.
Ethics oversight	The C57 mouse embryo (E11) frozen tissue sections were purchased from Zyagen, and this study did not involve other animals or other research organisms.

Note that full information on the approval of the study protocol must also be provided in the manuscript.
