# Supplementary figures and images for: Profiling of RNA-binding protein binding sites by in situ reverse transcription-based sequencing
Source: Nat Methods. 2024 Jan 10;21(2):247–58. doi: 10.1038/s41592-023-02146-w (PMC10864177; doi:10.1038/s41592-023-02146-w)

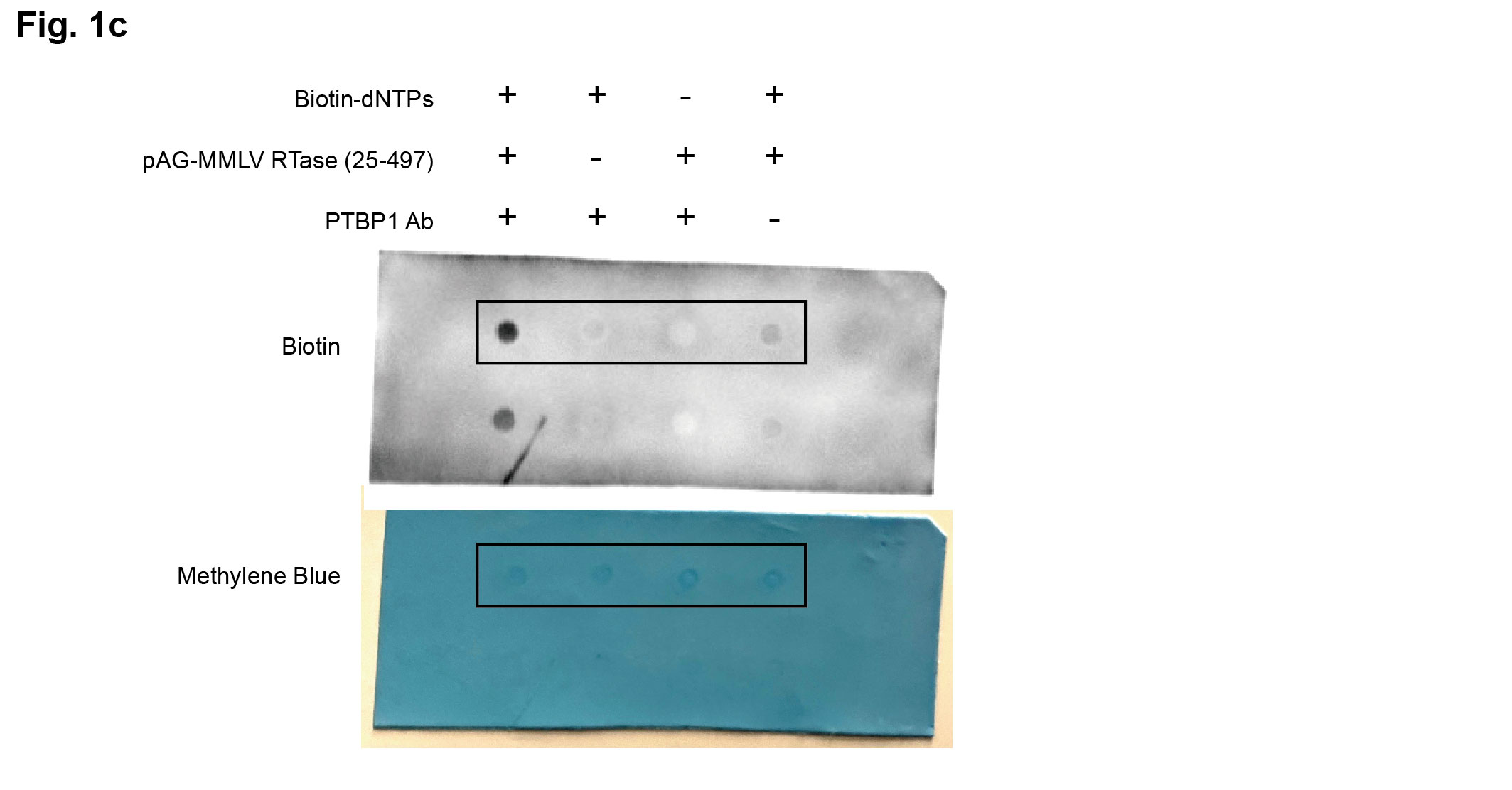

Supplement: Supplementary file 3 — Unprocessed dot blot. [file 41592_2023_2146_MOESM3_ESM.jpg]

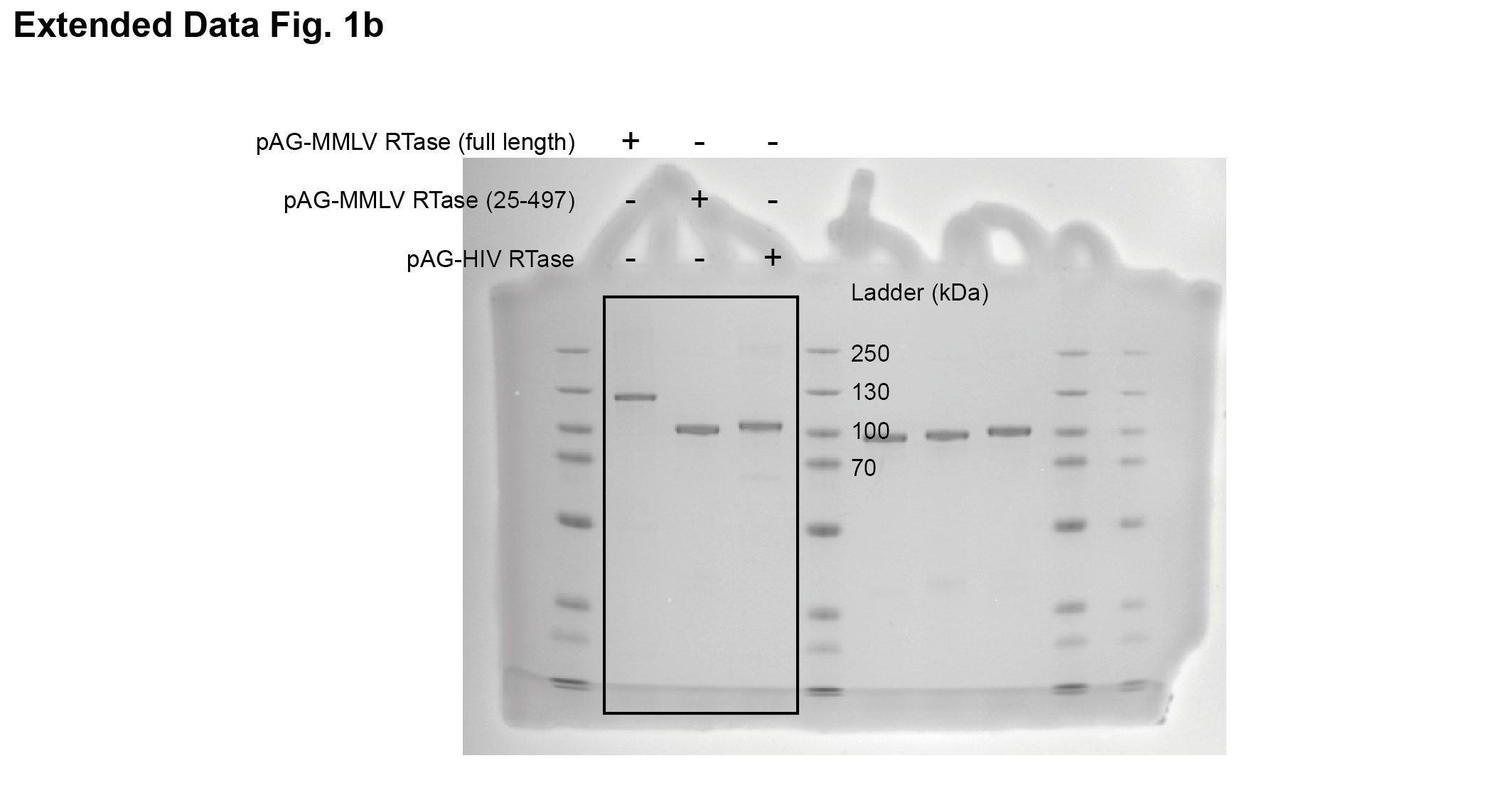

Supplement: Supplementary file 5 — Unprocessed gel. [file 41592_2023_2146_MOESM5_ESM.jpg]

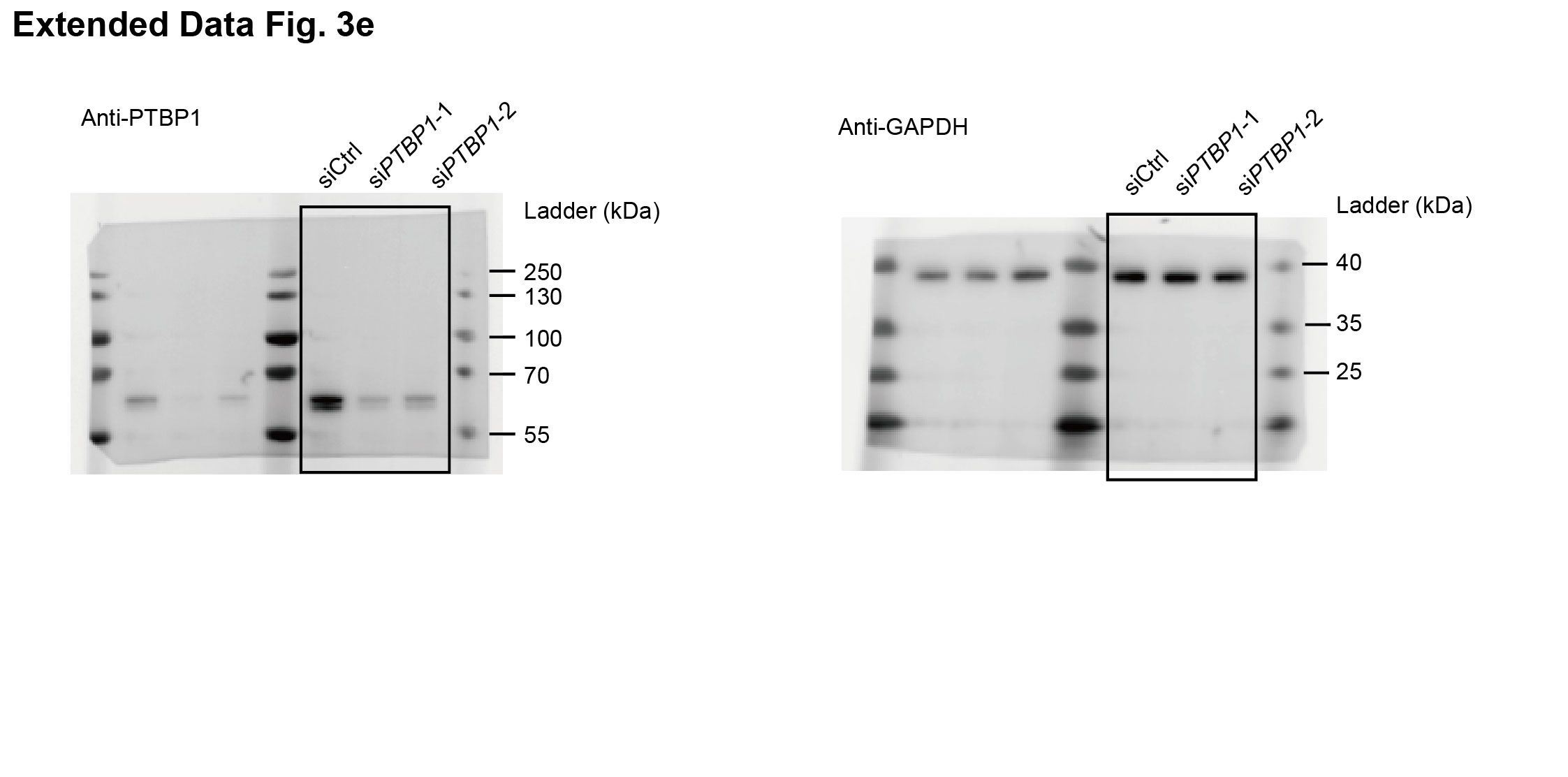

Supplement: Supplementary file 7 — Unprocessed western blots. [file 41592_2023_2146_MOESM7_ESM.jpg]

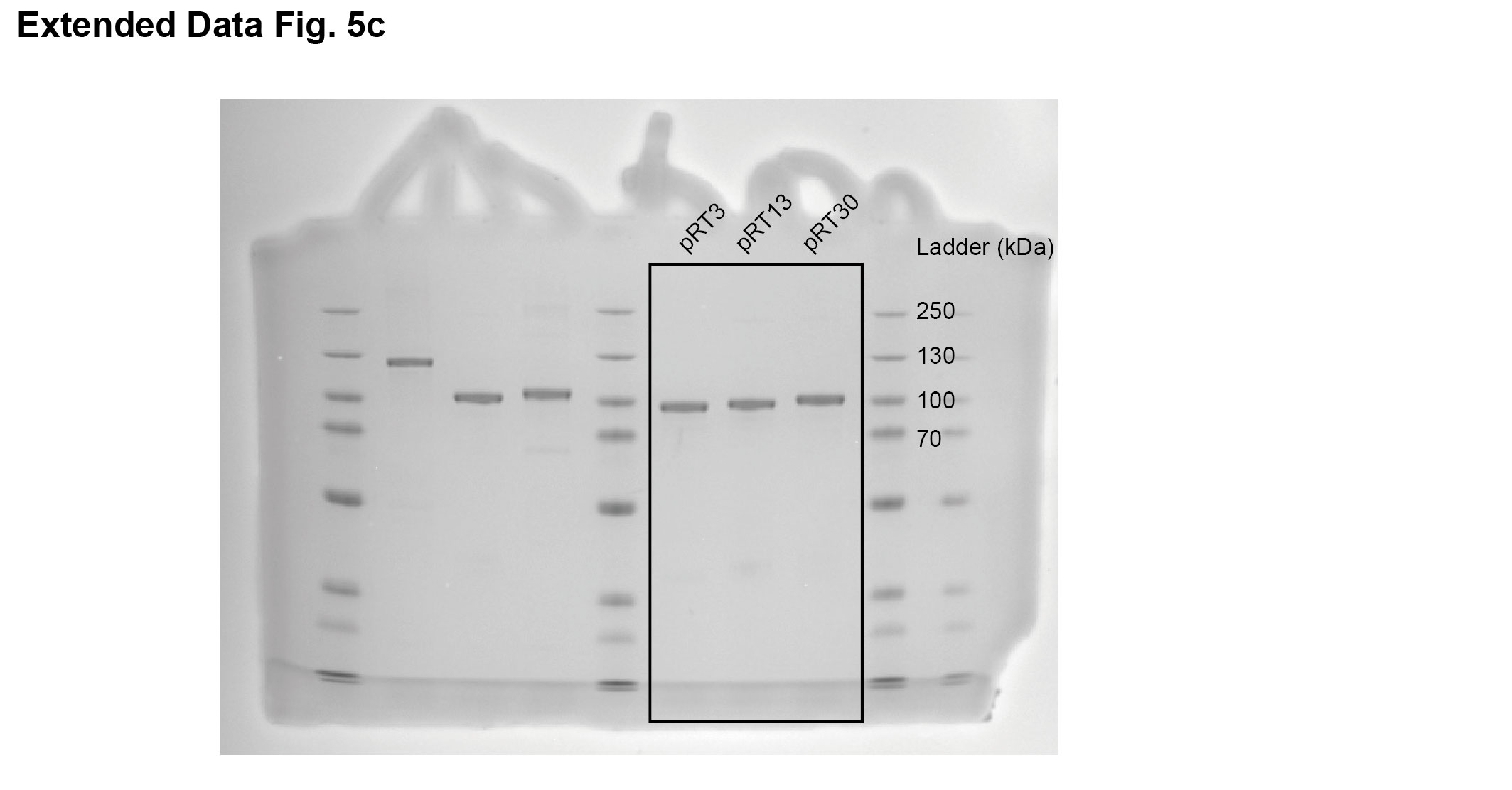

Supplement: Supplementary file 9 — Unprocessed gel. [file 41592_2023_2146_MOESM9_ESM.jpg]
